# A Mosaic of Future Maladaptation Predicted for the Widespread Tree *Nothofagus pumilio*

**DOI:** 10.1111/eva.70227

**Published:** 2026-03-27

**Authors:** Jill Sekely, Paula Marchelli, Maria Veronica Arana, Mario Pastorino, Ivan Scotti, Carolina Soliani, Lars Opgenoorth, Katrin Heer

**Affiliations:** ^1^ Eva Mayr Stihl Professorship for Forest Genetics, Albert‐Ludwigs Universität Freiburg Freiburg Germany; ^2^ Plant Ecology and Geobotany, Philipps‐Universität Marburg Marburg Germany; ^3^ INTA Bariloche, Instituto de Investigaciones Forestales y Agropecuarias Bariloche IFAB (INTA‐CONICET) San Carlos de Bariloche Argentina; ^4^ Institut National de Recherche Pour L'agriculture, L'alimentation et L'environnement (INRAE), URFM Avignon France

**Keywords:** genomic offset, maladaptation risk, *Nothofagus*, Patagonia

## Abstract

*Nothofagus pumilio*, or lenga beech, is a widespread and locally‐adapted tree species endemic to South America's Patagonia region. Its diverse populations span a 2000‐km‐long range in the Andes Mountains, which is already experiencing adverse effects from climate change. Here the Andes contain two uncorrelated temperature and precipitation gradients, which offers a unique opportunity to evaluate how gradients drive local adaptation and conversely how changes in these gradients could lead to future maladaptation risk. We predicted climate maladaptation risk for *N. pumilio* populations with genomic offset. Our dataset includes 493 adult trees in 20 natural forest sites across the distribution range that were sampled with a paired‐site study design, which creates the opportunity to also investigate genomic offset patterns at small spatial scales. Local climate data for both current and future projected conditions were extracted from the CHELSA online repository. We used 490 putatively‐adaptive single nucleotide polymorphisms, that is, those associated with chosen climatic gradients, and assessed genomic offset using two methods: LFMM ‘genetic.gap’ and Gradient Forest. We projected risk at sampled sites and spatially across the full Argentina species distribution range, considering three possible emission scenarios and two future time periods within the 21st century, using Ensemble Means calculated from CMIP6 projections. Contrary to our expectations, our results predict a complex mosaic of heightened maladaptation risk across the landscape, with particularly high values in northern treeline and southern valley populations, across all investigated scenarios. This suggests a more complicated pattern of risk than uniformly increased risk along elevation or latitude clines. Using external evidence, we contextualize our genomic offset results and investigate possible species' responses, including how maladaptation risk could impact Patagonian forests in the future.

## 1 Introduction

Patagonian forests are increasingly affected by climate change, which has already increased drought‐related dieback (Rodríguez‐Catón et al. 2016), increased wildfire (Kitzberger et al. 2022), and changed the limiting factors for tree growth (Reiter et al. 2024). Understanding the possible future consequences of climate change on Patagonian native tree species is a critical question, and its answer partially depends on populations' genetic compositions (Camus et al. 2024; Láruson et al. 2022). Forest tree populations tend to be locally adapted (Petit and Hampe 2006), which confers fitness advantages under local climate conditions. However, climate change threatens to turn those adaptations into maladaptation, and it is also likely that climate shifts will diverge across landscapes, meaning individual subpopulations could experience different levels of risk (Foden et al. 2019). The popular genomic offset method incorporates genomic information into future risk projections by calculating the weighted importance of environmental predictors on genetic structure (Láruson et al. 2022). However, recent evidence suggests that genomic offset analyses are sensitive to neutral demography and complexity of the adaptive environment (Gain et al. 2023; Láruson et al. 2022), so appropriate choice of study system and design are critical factors. The Patagonian Andes are a compelling case study for examining genomic offset because they have a pronounced north–south orientation with prevailing westerly winds, which creates two orthogonal climatic gradients. This provides a unique opportunity for disentangling individual climatic drivers of local adaptation and better informing possible future maladaptation.

One of the most widespread Patagonian forest tree species is *Nothofagus pumilio* (Poepp. & Endl.) Krasser, a locally‐adapted foundation species that is also increasingly threatened by climate change. This cold‐tolerant, deciduous species has a range that stretches over 2000 km in the Andes mountain range (36°S–56°S, Veblen et al. 1996). Here, the Andes contain uncorrelated climate gradients, namely a north–south temperature and day length gradient and an east–west precipitation gradient. Genetic diversity of *N. pumilio* also varies across this landscape. Population structure is mainly oriented along the latitude axis (Mattera et al. 2020; Sekely et al. 2024), likely due to post‐glacial expansion patterns (Soliani et al. 2015). Greater genetic diversity has been reported in both northern populations (Mattera et al. 2020; Sekely et al. 2024), and some low‐elevation forests within local elevation gradients (Mathiasen and Premoli 2013). The presence of local adaptation is supported by empirical evidence from transplant experiments and landscape genomics analysis. Transplant experiments using materials from elevation gradients (i.e., as proxy for temperature) found genetically‐controlled adaptive traits in various traits such as branching morphology (Soliani and Aparicio 2020), leaf morphology (Mathiasen and Premoli 2016), and phenology (Premoli et al. 2007). Along precipitation gradients, drought‐related adaptations have been identified, including water use efficiency (Soliani et al. 2021), growth under drought stress (Ignazi et al. 2020), and stomatal control (Diaz et al. 2022). Precipitation and temperature gradients were also recently investigated in a landscape genomics study, and signatures of selection were identified among candidate genes related to cold, heat, drought, and immune response (Sekely et al. 2024). While local adaptation can confer fitness advantages to local conditions, mounting evidence suggests that climate change has already affected *N. pumilio* populations and could be driving maladaptation.

Reduced precipitation and increased temperature have already been observed over the past century in the Patagonian Andes (Barros et al. 2015). These changes are associated with *N. pumilio* fitness reductions, including increased dieback (Tarabini et al. 2021), reduced seedling establishment (Aschero et al. 2022), and lower forest vitality (Rodríguez‐Catón and Villalba 2018). Critically, both climate change patterns and effects can be spatially divergent. For example, contrasting growth trends have been observed at adjacent mesic and dry treelines of *N. pumilio* under warming conditions (Brand et al. 2022), demonstrating that individuals and subpopulations can respond differently even to similar climate changes.

Despite this documented spatial heterogeneity in climate change and its effects, as well as the genetic diversity of *N. pumilio* (Soliani et al. 2015) and many other Patagonian species (Sérsic et al. 2011), the region is lacking genomics‐informed climate change risk assessment studies. To our knowledge, only one other genomic offset analysis has been conducted in Patagonia, which focused on the rare conifer species *Araucaria araucana* (Varas‐Myrik et al. 2022, 2024). For a more comprehensive view of how climate change might impact Patagonian forests, genomics‐informed risk assessments should also be performed for widespread species that define the local ecosystems such as *N. pumilio*. Here, we use the genomic offset method to measure the mismatch between current allele frequencies and those that might be required for survival under future climatic conditions (Capblancq et al. 2020; Rellstab et al. 2021).

We applied genomic offset to a high‐confidence single nucleotide polymorphism (SNP) dataset of *N. pumilio* adults sampled across its main distribution range in Argentina (Sekely et al. 2024). A paired‐site sampling design incorporated local elevation gradients, which provides a unique opportunity to investigate offset at large and small spatial scales, and along latitude and elevation gradients. We expected that (i) latitude range edges would have greater offset while core areas would have less (e.g., Gougherty et al. 2021) and (ii) locally higher‐elevation sites would have greater offset than lower (e.g., Lachmuth et al. 2024). By predicting climate change impacts on *N. pumilio*, and identifying areas that may have high maladaptation risk, we aim to safeguard the continued health of Patagonian forest ecosystems.

## 2 Materials & Methods

### 2.1 Sampling Design and Genetic Dataset

Our SNP dataset comes from 493 dominant adult *Nothofagus pumilio* sampled across 20 natural forest sites in the Argentinean Andes (Sekely et al. 2024). Briefly, authors used a paired site sampling design to both maximize climatic distance and minimize neutral genetic distance, with the aim of differentiating between climate‐adaptive and neutral genetic variation (Lotterhos and Whitlock 2015). Sites within each pair were an average of 1.1 km linear distance apart (range 0.6–2.2 km) and had an average elevation difference of 230 m (range 150–320 m). Targeted sequencing was performed for 2183 candidate genes related to fitness traits of interest, including stress response, growth, and phenology (Milesi et al. 2024; Sekely et al. 2024). Sequenced DNA was aligned with a *N. pumilio* de novo transcriptome (Estravis‐Barcala et al. 2021). After quality filtering, which included pruning for linkage disequilibrium and paralogs, the dataset contained 47,336 SNPs within 1632 contigs (i.e., coding regions along the genome). We started our analysis with this quality‐filtered dataset, and first calculated some additional genetic diversity statistics, namely locally common alleles and private alleles within each sampling area. Allele frequencies were calculated using R::hierfstat ‘allele.count’ (v. 0.5.11, Goudet and Jombart 2022) and base R commands (R version 4.5.0, R Core Team 2025). Alleles were considered locally common if their frequencies were at least 5% in one sampling area and less than 5% in all others. Private alleles were calculated with R::poppr “private_alleles” (v. 2.9.7, Kamvar et al. 2025). Finally, for the genotype‐environment association and genomic offset analyses, we removed very rare alleles from the dataset by applying a minor allele frequency threshold of 5%.

### 2.2 Past Climate Data and Genotype‐Environment‐Associations

Climate data were extracted from the online repository CHELSA v 2.1 (Karger et al. 2017, 2018). The “past” dataset contains averaged annual data for the reference period 1981–2010. We chose five variables that are likely drivers of *N. pumilio* selection (see Lind and Lotterhos 2025) related to temperature and precipitation: temperature isothermality (“bio3”), mean growing season temperature (gst), snow cover days (scd), total annual precipitation (bio12), and precipitation seasonality (bio15) (Figure S1). Chosen climatic variables had relatively low Pearson's correlations among themselves (*R* < 0.77, Figure S2), which is a critical prerequisite for GEA studies to avoid confounding (Rellstab et al. 2015). We extracted climate data at individual tree positions in R using the terra::extract() (v 1.8–60, Hijmans 2025) and bilinear option, which interpolates values from the four nearest raster cells, to approximate finer‐scaled data than CHELSA native resolution (i.e., 30 arc‐sec, ~1 km^2^). Extracted climate data were scaled before GEA analysis.

Some studies have shown that analyzing only putatively adaptive SNPs can improve genomic offset results (e.g., Gain et al. 2023), so we subset our starting SNP list using genotype‐environment association (GEA) analysis. A previous GEA analysis on this SNP dataset (Sekely et al. 2024) identified 445 SNPs with allele frequencies that were significantly associated with temperature, precipitation, or day length parameters, and we used this as a starting list. However, we used slightly different climate parameters for the genomic offset analysis, due to parameter availability in the future projected datasets, so we ran a supplemental GEA analysis to add any SNPs that were significantly associated with the updated climate parameters. For this we used the latent factor mixed model (LFMM, Jumentier 2021) GEA method, since we also used the LFMM‐based genomic offset method (genetic.gap). We used *K* = 3 based on prior analysis (Sekely et al. 2024). We applied a false discovery rate threshold of 1% to reduce false‐positive associations (François et al. 2016). As a final note, common practice in GEA‐focused studies is to retain only high‐confidence candidate loci that are identified by more than one test, since the aim is usually to identify SNPs under selection (Rellstab et al. 2015). However, our aim with the genomic offset analysis was to select putatively adaptive SNPs (Lind and Lotterhos 2025) and weigh the importance of environmental variables on their frequencies (Láruson et al. 2022) so instead we chose a lenient approach and retained all candidate loci that were identified by at least one method in either study (e.g., Chen et al. 2024).

### 2.3 Future Climate Data

We combined five CMIP6 global circulation models into an Ensemble Mean to increase reliability and decrease bias of individual models (Ahmed et al. 2019; Wang et al. 2018). The CHELSA algorithms (Karger et al. 2017) have been applied to the following pre‐selected models: GFDL‐ESM4, MPI‐ESM1‐2‐HR, MRI‐ESM2‐0, IPSL‐CM6A‐LR, and UKESM1‐0‐LL. Independent studies have assessed these models for accuracy in Patagonia by comparing recorded empirical data against historical simulations and suggest that they are appropriate choices for the region (Ortega et al. 2021; Reboita et al. 2024). Of note is that UKESM1‐0‐LL is considered a “hot model” (Cannon 2024), but this model has been shown to realistically reflect dry‐season rainfall amounts in central Chile (Ortega et al. 2021). Ensemble Mean rasters were created in R with terra::mean(), then future climatic data for each tree's location were extracted from the new rasters using the same method as for past data.

We considered three emission scenarios for two future time periods. Shared socio‐economic pathway (SSP) scenarios combine possible levels of future greenhouse gas emissions and socioeconomic change to approximate the severity of future warming. The plausibility of individual scenarios is inherently unknowable (Huard et al. 2022; Kundzewicz et al. 2018) and scenarios have been identified as a great source of uncertainty in genomic offset calculations (Gain et al. 2023; Lind et al. 2024). Therefore, we assessed a range of scenarios rather than averaging multiple SSPs or choosing a single scenario (e.g., Foden et al. 2019). We used three pathways: (a) optimistic scenario (SSP 1–2.6), (b) moderate scenario (SSP 3–7.0), and (c) pessimistic scenario (SSP 5–8.5). Finally, we ran the analyses for two future time periods: (1) 2041–2070 (hereafter called “mid‐century”) and (2) 2071–2100 (“late‐century”).

### 2.4 Genomic Offset Methods and Considerations for Population Structure

We calculated genomic offset values using two independent methods: LFMM “genetic.gap” (hereafter called LFMM) and Gradient Forest. Previous studies have shown correlations between common garden fitness offsets and LFMM offset (Gain et al. 2023) and Gradient Forest offset (Láruson et al. 2022). First, the LFMM method estimates regression coefficients between environmental variables and allele frequencies and then calculates genomic offset as the weighted distance between past and future environment, where the weights reflect the influence of each environmental variable on genetic structure (Gain et al. 2023). Second, Gradient Forest is a non‐parametric multivariate machine learning approach (Fitzpatrick and Keller 2015) with the unique ability to detect non‐linear trends and characterize compositional turnover in allele frequencies. LFMM and Gradient Forest both calculate a genomic offset value for each individual tree genotype, and we averaged these values *post hoc* to get mean values per sampling site. We ran both methods for all three SSP and two future time periods. Following our GEA analysis, we retained 490 SNPs for offset analysis.

*Nothofagus pumilio* genetic population structure is predominantly latitude‐oriented (Mattera et al. 2020; Sekely et al. 2024; Soliani et al. 2012), which means it is collinear with latitude‐oriented climatic gradients (Figure S2). Collinearities can confound both GEA and genomic offset analyses (Fitzpatrick et al. 2021; Gain et al. 2023; Rellstab et al. 2021). The LFMM “genetic gap” analysis natively incorporates population structure by calculating latent factors. However, Gradient Forest does not address structure, so we added it as a confounding variable using the method developed by Gain et al. (2023). For both analyses, the first step was to run the LEA::lfmm2() command (v. 3.20.0, Frichot et al. 2025) and compute latent factors for *K* = 3. For LFMM, the “genetic.gap” command was subsequently run. For Gradient Forest analyses, we used the gradientForest R package (v. 0.1–37, Smith et al. 2023) and wrapper functions (“go_gf” and “gf_pred”) around the predict.gradientForest() function (see Gain et al. 2023) to use those same latent factors as confounding effects (Figure S3). We ran 500 regression trees per SNP. Finally, linear regressions between genomic offset values and geographic gradients, namely latitude and elevation, were quantified with the ggpmisc package (0.6.2, Aphalo 2025) and stat_poly_eq() command. All analyses were run in the RStudio environment (R Core Team 2025). Code and inputs used for running the analysis can be found at: https://github.com/jtsekely/Nothofagus_genomic_offset.

We also spatially projected offsets across the species' current distribution in Argentina alone, since all samples were obtained there. For LFMM, first the difference between future environment and past environment was calculated for the current species distribution range. Each environmental predictor was then individually weighted based on effect sizes (i.e., from the previous analysis of sampled trees), and values were averaged to give the final genomic offsets (Gain et al. 2023). For Gradient Forest, we used the run_gf() and gf_pred() functions written by Gain et al. (2023) to train a model that incorporated latent factors (genetic structure) as confounding factors. This model was then used in the gradientForest::predict() command, first with past environmental data and then with each scenario's future environmental data. The Euclidian distance between past and future values was calculated to result in the genomic offset values. Code for mapping was based on Fitzpatrick and Keller (2015) and modified to work with the terra package.

Raster outputs were post‐processed in QGIS (v 3.40). As a final note, models often have decreased accuracy when used to predict values in places with novel conditions in relation to training data (e.g., Fraslin et al. 2022), for example if there are climate characteristics not existing anywhere within the current global distribution range (DeSaix et al. 2022; Lind and Lotterhos 2025). Therefore, we predominantly used this spatial projection method to provide a broad‐stroke picture of how maladaptation patterns may look across the current distribution range.

## 3 Results

### 3.1 Genetic Diversity and Candidate SNPs

Each sampling area had between 153 and 1631 locally common alleles (i.e., > alleles with 5% frequency in that population alone), and 672–5392 private alleles (i.e., total alleles found in that population alone, irrespective of frequency) (Table S1). The northernmost area, Epulaufquen, had the greatest number of both, with decreasing values towards southern locations. Notably, locally common alleles were more evenly distributed along the latitude range than private alleles. The GEA analysis identified 45 supplemental SNPs that were associated with at least one of the five chosen environmental parameters (Table S2) and were not already included in the starting candidate SNP list of 445 SNPs. Thus our starting SNP set contained 490 SNPs.

### 3.2 Geographic Patterns in Genomic Offset Values

Offset values cannot be directly compared across offset methods (Gain et al. 2023), so we assessed trends and patterns rather than absolute values between the two methods. Gradient Forest and LFMM results generally showed similar trends, suggesting the genomic offset results are robust. We present Gradient Forest results in the main manuscript, and complementary LFMM figures can be found in Appendix S1.

#### 3.2.1 Genomic Offset: At Sampled Sites

**FIGURE 1:**
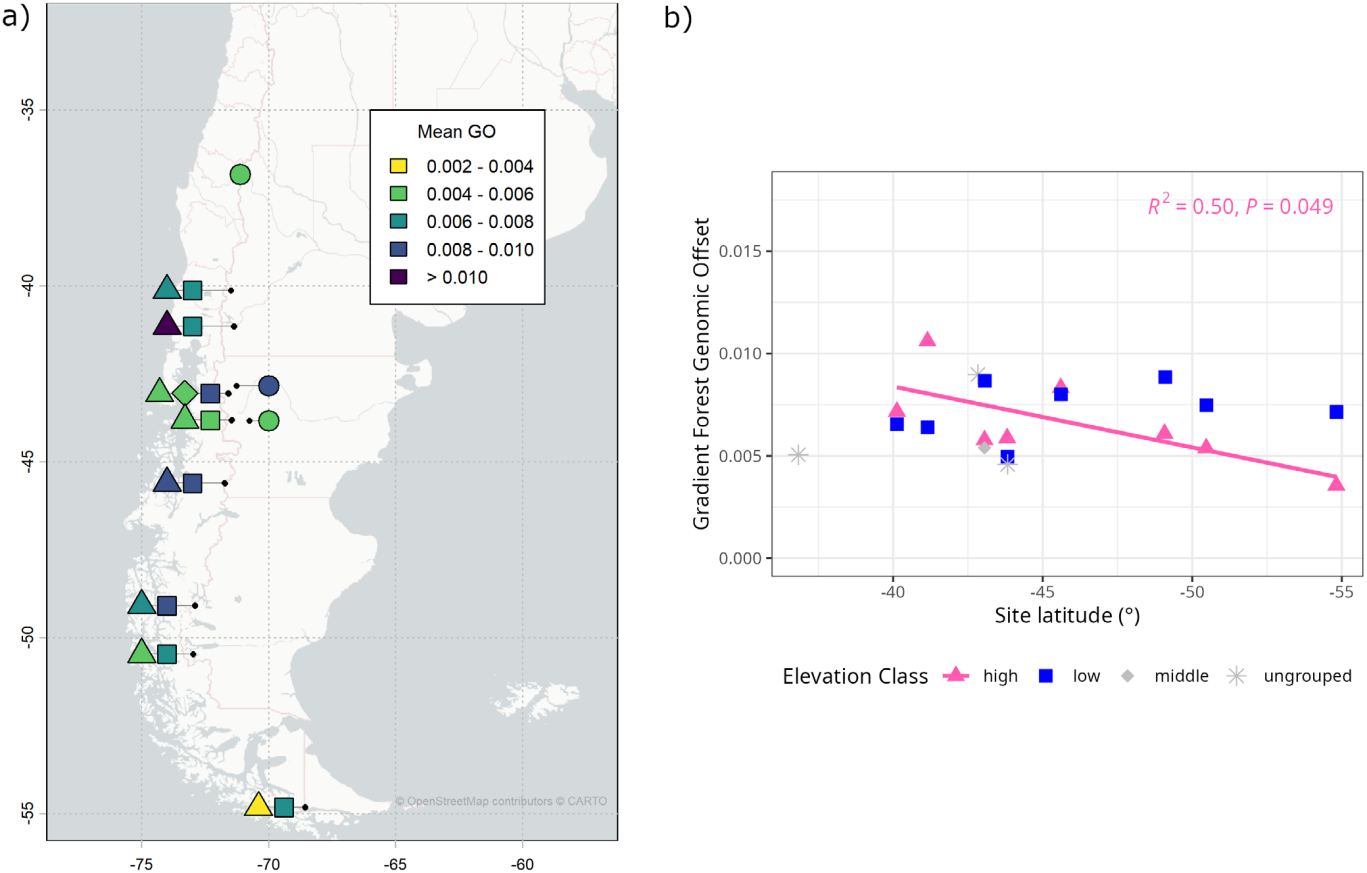
Mean Gradient Forest offset values of all trees per sampling site and relationship with site latitude for one time period (2041–2070) and a moderate emissions scenario (SSP 3–7.0). (a) Map showing values at sampled sites. Colors are binned for readability. Black dots indicate real locations of sampling sites, and points are jittered longitudinally (but not latitudinally. Basemap created with maptiles R package (Giraud et al. 2025, background maps OpenStreetMap contributors CARTO). Real values are shown in b), where relative elevation class is indicated by point shape (■ = low, ◆ = middle, ▲ = high, ❋ = ungrouped) as well as color for pattern clarity (pink = high, blue = low, grey = middle and ungrouped). Solid regression line indicates a significant relationship (*p* ≤ 0.05) and missing lines indicate no meaningful relationship (*p* > 0.1). Line color matches elevation class (pink = high elevation; the low‐elevation correlation was insignificant so line was omitted). *R* ^2^ and *p*‐value of shown correlation are at top right. For the other time period × emission scenario graphs, see Figure S4.

*Nothofagus pumilio* has a long latitude distribution and is predominantly a montane species, so to characterize spatial trends, we focused on latitude and local elevation gradients. Mean genomic offset values at the locally higher‐elevation sampling sites had a negative relationship with latitude, meaning the values were greatest in the north and least in the south (Figure 1, Figure S4, LFMM results in Figure S5). In all mid‐century scenarios, these correlations with latitude were significant and fairly strong (*R* ^2^ > 0.5, *p* ≤ 0.05), while in late‐century, these correlations were weaker and only moderately significant (*R* ^2^ < 0.45, *p* ≤ 0.1), with the exception of the highest emission scenario. In contrast, values at locally lower sampling sites had no significant relationships with latitude across scenarios (all *p* > 0.1). Looking within sampling site pairs (i.e., within local elevation gradients), trends also varied by latitude (Figure 1b, Figure S6). Namely, site pairs in northern (36°S–42°S), central (42°S–49°S), and southern (49°S–56°S) regions exhibited different patterns. In the two northern pairs (San Martin de los Andes (40.1°S) and Cerro Otto (41.1°S)), offset values were consistently greater at the higher elevation sites than the lower. In central pairs, the greater offset value fluctuated between the locally high and low sites across scenarios. In the south, the southernmost pair (Ushuaia, 54.8°S) always had greater offset in the lower‐elevation site (Figure 1, Figure S5), and the other southern site pairs (El Chaltén and El Calafate) had greater offset values at lower elevations in all scenarios except moderate and pessimistic emission scenarios during the late century. Finally, relationships between genomic offset and latitude only (i.e., ignoring local elevation class) were mostly insignificant (Figure S5), although two late‐century scenarios showed weakly negative relationships (SSPs 1–2.6 (*R* ^2^ = 0.14, *p* = 0.100) and 5–8.5 (*R* ^2^ = 0.20, *p* = 0.047)). Thus, northerly sites had slightly greater offsets overall than the central or southern sites.

#### 3.2.2 Genomic Offset: Spatial Projections

**FIGURE 2:**
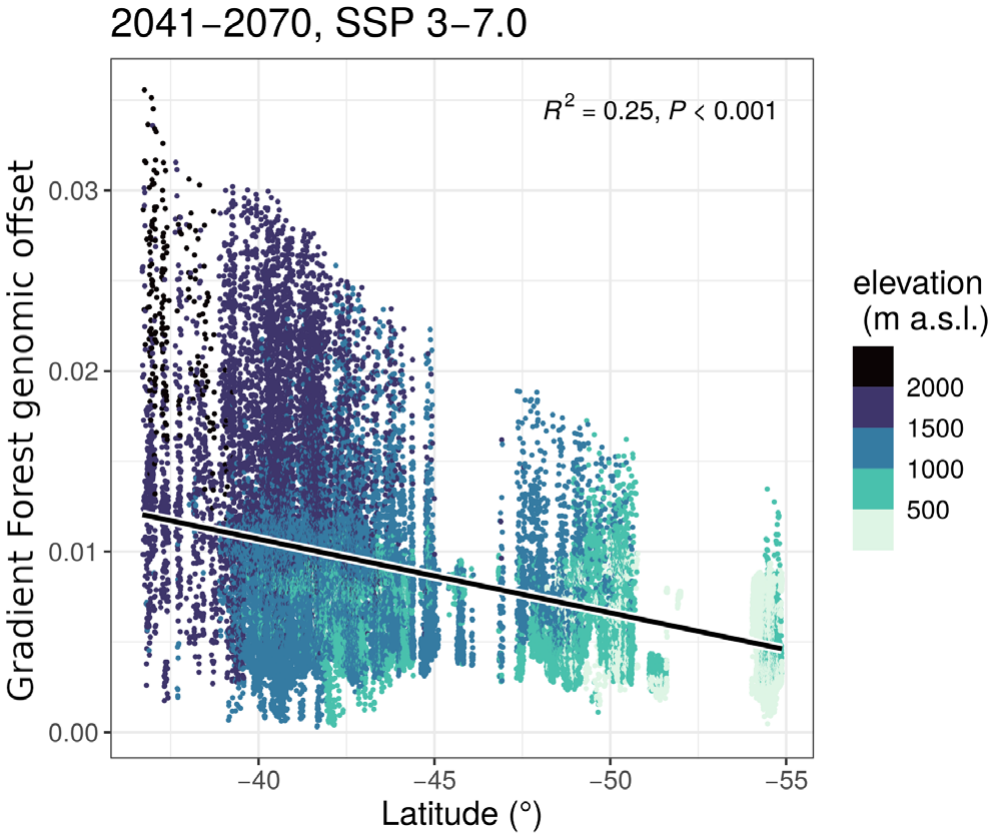
Gradient Forest genomic offset values per raster cell for the full Argentina species distribution in relation to latitude and elevation. Plot is for the mid‐century (2041–2070) and moderate emission (SSP 3–7.0) scenario. Color indicates binned elevation class, in meters above sea level. For other scenarios, see Figure S7.

**FIGURE 3:**
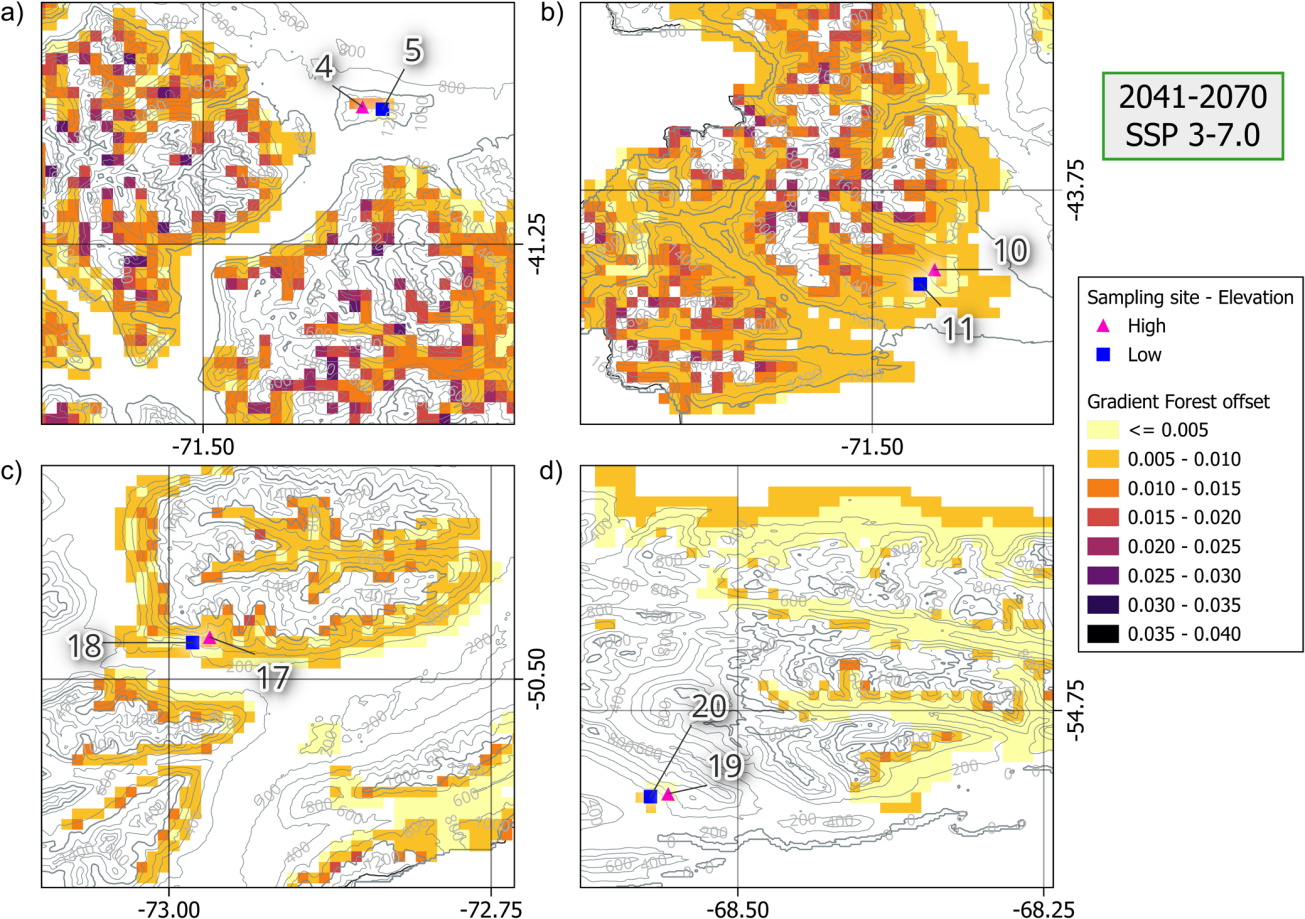
Spatial projection map of Gradient Forest genomic offset values for the mid‐century (2041–2070) and moderate‐emission scenario (SSP 3–7.0) for four representative areas of the *Nothofagus pumilio* range in Argentina. The shown areas each contain one sampling site pair, from north to south: (a) Cerro Otto (sites 4 & 5), (b) Lago Guacho (10–11), (c) El Calafate (17–18), and (d) Tierra del Fuego (19–20). Shapes indicate the elevation class of each site: High (▲) or low (⬛). Color of each 1‐km‐square pixel indicates the binned, projected Gradient Forest genomic offset value, from low (yellow/orange) to high (purple/black). Elevation isohypses indicate local topography, with thicker lines shown at 1000 and 2000 m a.s.l and thinner at every 200 m in between.

These latitude and elevation trends became more pronounced when offset was spatially projected across the current distribution range in Argentina. Over all 6 scenarios, range‐wide offset values had significant but weak negative relationships with latitude (*p* < 0.001, *R* ^2^ < 0.3), meaning the greatest values are towards the north (Figures 2 and 3, Figures S7–S13). However, it is important to note there are some low offset values at northern latitudes as well, typically at lower elevations (Figures 2 and 3). Spatial projections also show greater ranges in values along local elevation gradients (i.e., at similar latitudes) than results from sampled sites (Figures 1 and 2). A notable departure from the sampling‐site results is that higher elevations across the range usually exhibited the greatest values, also in most central and southern locations (Figures 2 and 3, Figure S9). An exception is in the southernmost reaches of Tierra del Fuego, where locally greater offsets were sometimes found near sea‐level (Figure 3d, Figure S12). As a final remark, elevation and latitude are autocorrelated within the species' range, meaning populations in the north grow at higher elevations than those in the south (Figure 2). Together, both the sampled‐site and spatial projection results suggest a non‐clinal mosaic of genomic offset values across the landscape, influenced by the interaction between local elevation and latitude, rather than monotonic gradients wherein higher or lower elevation forests always have greater offset values.

## 4 Discussion

### 4.1 Maladaptation Risk Is a Mosaic Across Landscapes

*Nothofagus pumilio* is a widespread Patagonian tree species that exhibits evidence of local adaptation, and predicting its maladaptation risk under future climate change is a critical challenge for local scientists and foresters alike. Using two genomic offset methods, we predicted there will be a non‐clinal mosaic of maladaptation risk across its contemporary distribution range. Offset values at sampled sites indicated that there is high risk possible at many points along the elevation and latitude ranges, notably including high elevations in the north and low elevations in the north. Meanwhile, spatial projections of the full Argentina distribution range showed the greatest overall offset values might be found at the northern trailing edge. Together, these results suggest that maladaptation risk may be complex in *N. pumilio* , rather than adhering to straightforward clines of increasing maladaptation risk with decreasing elevation and/or latitude.

### 4.2 A Brief Word About Interpretation

Genomic offset is a developing method, and there is ongoing discussion about how best to interpret its values (Láruson et al. 2022; Gain et al. 2023; Lachmuth et al. 2024; Lotterhos, 2024), so here we briefly describe our interpretation approach to guide the following Discussion. First, we interpreted genomic offset as a proxy for potential maladaptation risk, and therefore we expected negative relationships between genomic offset values and fitness (Capblancq et al. 2020; Lind et al. 2024). Second, genomic offset values are inherently positive, so we interpreted relative magnitude as relative risk.

### 4.3 Predictions Challenge Monotonic Risk Gradients

The absence of a strong significant relationship between genomic offset and latitude at sampled sites suggests that *N. pumilio* subpopulations at central latitudes could be at similar risk levels as those closer to the northern or southern edges, contradicting our first prediction. While range edges were traditionally hypothesized to be at greater risk of climate change effects than central (Jump et al. 2006), increasing evidence suggests that central areas may be just as vulnerable as edges, for example in the related European species *Fagus sylvatica* (Cavin and Jump 2017; Hacket‐Pain et al. 2016). Our results may support the latter, also in conjunction with *N. pumilio* fitness changes that have been observed along the range, which we discuss below. On the other hand, our spatial projections did predict a significant (but weakly) negative relationship between offset and latitude, and the greatest average values were consistently found at lower‐latitude trailing edges, providing partial support for our prediction (i). This is likely due to the greater predicted environmental change at lower latitudes. Critically, offset values in some areas were thrice the maximum value predicted for any sampled site under the same CMIP6 projection(s), and any projections beyond training data must be handled with caution (Lind and Lotterhos 2025). However, these higher genomic offset values outside the sampled area are likely reflecting greater predicted environmental change at those locations, which will nevertheless impact future evolution of the species.

When local elevation clines are also considered along the latitude gradient, we also found complex patterns. Both the sampled‐site and spatial projection results identified possible risk hotspots at high elevations in northern and central latitudes, and some lower elevation locations in the southern latitudes. Forests at warmer range edges (e.g., low elevations) are predicted to experience greater drought‐ and heat stress in the near future (Hampe and Petit 2005), and this informed our study prediction (ii), but this prediction was only partially supported by our results. One explanation for high‐elevation but low‐latitude hotspots could be elevation‐dependent warming, where high elevations are projected to warm faster. This pattern has already been observed in the tropical Andes (Pabón‐Caicedo et al. 2020), and CMIP6 projections predict it will occur in nearby Antarctica (Zhu et al. 2024). This is also critical point for the methodology, since locations with greater predicted differences in selective climate parameters, including temperature, will also show greater genomic offsets. A final note regarding the elevation‐latitude relationship in *N. pumilio* is that the two are strongly correlated within the species' range, meaning populations in the north grow at higher elevations than those in the south (e.g., above 1000 m a.s.l. at 36°S, and between 0 and 600 m at 55°S). This geographic autocorrelation made it difficult to determine whether offset patterns were more closely related to latitude, elevation, or a combination thereof.

### 4.4 Linking Offset Results to Fitness Changes and Risk Modeling

The relationship between climate change and fitness change is a complex one (Clark et al. 2024), which also has bearing upon genomic offset result interpretation. Climate change has already impacted fitness of *N. pumilio* across its range, but contrasting effects have been found even at similar latitudes and elevations, suggesting fitness changes are also a mosaic. For example, one study in the north (38°S–43°S) found that severe droughts following wet periods triggered a lasting radial growth decline in most large *N. pumilio* individuals, but other individuals in the same forests showed a growth increase (Rodríguez‐Catón et al. 2016), possibly due to reduced intraspecific competition or unobserved genetic differences. Low‐elevation and drier high‐elevation forests can show slight radial growth decreases in the north (Reiter et al. 2024), but curiously, upper montane forests have shown stable or increasing growth in past decades in both northern (41°S–42°S, Reiter et al. 2024) and southern (45°S–47°S, Gibson‐Carpintero et al. 2022) locations. Furthermore, negative effects of drought on establishment at low elevations have also been reported, while positive effects were actually observed at higher elevations (Aschero et al. 2022). These diverse fitness changes suggest that our predicted genomic offset patterns may not be entirely unexpected, but also that the highest genomic offset values may not directly translate to the greatest fitness decreases, since many other factors beyond macroclimate could play a role.

Our genomic offset trends are also generally supported by other recent risk assessment studies of *N. pumilio*. Two independent studies reported similar predicted trends along latitude and elevation gradients, including great loss of suitable habitat for *N. pumilio* in coming decades. A bioclimatic modeling study, using climatic data in northern latitudes, predicted a sharp decline in suitability of high‐elevation sites (Fierke et al. 2024). An ecological niche modeling study incorporated neutral diversity data across the full range of multiple species, and predicted the most severe habitat losses for *N. pumilio* at higher elevations in the north, especially between 39°S and 42°S (Soliani et al. 2024). Notably, the latter study also found high risk of habitat loss at both Tierra del Fuegian low elevations and local treelines. Our study provided an extra dimension to these results by weighing climate parameters by their relative importance for adaptive genetic diversity. The greater maladaptation risk identified in the present study and higher habitat loss at the same locations could be compounding threats, since decreased fitness mixed with habitat loss could create a particularly dire situation for local populations.

### 4.5 Possible Ramifications for Patagonian Forests

Genomic offset predictions can be used to predict maladaptation risk but not populations' future responses (Capblancq et al. 2020), which depends on more complex processes (Shaw 2019). To put these genomic offset results into context and determine what they could mean for Patagonian forests, we considered possible responses to future climate change using external evidence. Further adaptation could allow populations to keep pace with climate change velocities (Foden et al. 2019), and populations with higher genetic diversity usually have greater adaptation potential (Forester et al. 2022; Hampe and Petit 2005). Genetic diversity is greatest in northern *N. pumilio* populations and decreases poleward (Mattera et al. 2020; Sekely et al. 2024), which was also supported by our private allele results, although we also found that southern areas still contain many locally‐common alleles that are rare or absent in other areas. This genetic diversity may contribute to mitigation through future adaptation, even in locations with high predicted offset. Meanwhile, some higher‐elevation populations of *N. pumilio* have also been shown to contain lower genetic diversity levels (Mathiasen and Premoli 2013; Premoli 2003), and while it's critical to mention that our sampling strategy did not include treelines, this could mean the populations at treeline risk hotpots, as identified by our spatial offset projections, have reduced diversity as well. Finally, plasticity could also be an important adaptation strategy, and it has already been observed in *N. pumilio* seedlings under warming conditions (Aparicio et al. 2025; Torres et al. 2025).

A location's future habitability is not the same as the predicted maladaptation risk for current inhabitants, and it is possible that some locations will become more habitable for foreign populations even if the local population declines. This may already be occurring, for example, in forests where some trees have experienced growth increases even as adjacent trees declined (Rodríguez‐Catón et al. 2016). Natural gene flow from more warm‐adapted populations is one possible mitigation strategy (Vitasse et al. 2021), although *N. pumilio* gene flow tends to be spatially limited (Soliani et al. 2025), and therefore increased human‐assisted migration may be necessary (Pastorino et al. 2021). At the same time, lower elevations are usually dominated by local populations of other less cold‐tolerant *Nothofagus* species, where *N. pumilio* is outcompeted (Cagnacci et al. 2020). Those species may experience range shifts as well, which could increase interspecific competition along the elevation trailing edges (Paquette and Hargreaves 2021). Thus, although lower‐elevation populations in northern latitudes did not have especially high local offset values, non‐climate factors could nevertheless cause forest decline. A possible consequence is that the *N. pumilio* elevational distribution range will contract. This highlights the need to predict climate change impacts on other *Nothofagus* species, for a more comprehensive overview of Patagonia's future.

The only other known Patagonian genomic offset study concerns the rare conifer species *Araucaria araucana* (Varas‐Myrik et al. 2022, 2024), which has remnant populations in northern Patagonia. That study predicted the greatest genomic offset values at the lowest local elevations in Chile, which is opposite from our predicted trend for *N. pumilio* at the same latitudes in Argentina. This highlights three points. First, it is necessary to assess genomic offset for individual species in the same locations to attain a comprehensive overview of how local forest ecosystems might be affected by climate change, since different adaptive genes and climatic pressures may be relevant for each. Second, habitat suitability changes will likely differ among species. For example, the distribution modeling study that considered *N. pumilio* also assessed *A. araucana*, and results suggested that climate change could render 100% of *A. araucana* 's most genetically diverse populations' locations unsuitable by 2070 (Soliani et al. 2024). Third, these divergent findings could indicate that the species' ranges will move independently of each other and even form new species assemblages in the future, which has also happened in the past (Webb III and Bartlein 1992; Williams et al. 2004). The combination of suitable habitat loss with high maladaptation risk, which has now been identified in multiple species, may be particularly problematic for Patagonian forests.

## 5 Conclusion

The widespread and locally‐adapted Patagonian tree species *Nothofagus pumilio* is likely to face many challenges under climate change, including maladaptation risk. Using two genomic offset methods, we forecast intraspecific risk for different populations along the species' distribution range across various emission scenarios and time periods. Our results suggest there will be an intricate mosaic of risk across the range, rather than monotonic clines of increasing risk with decreasing latitude and/or elevation. Some areas, including northern treelines and southern valleys, presented especially high offset values, which suggests these populations may be at greater risk, particularly in populations that currently have reduced diversity. Our mosaic‐like results for genomic offset are also reflected in diverse fitness and climate changes that have already been observed across the range, and together with other bioclimate modeling studies, they suggest that the future of *Nothofagus pumilio* forests is uncertain.

## Conflicts of Interest

The authors declare no conflicts of interest.

## Supporting information


**Figure S1:** Average annual climate conditions for each tree within each of the 20 sampling sites for *Nothofagus pumilio* based on CHELSA v2.1 for the reference period 1981–2010. Variables are (a) annual precipitation (shorthand: bio12), (b) precipitation seasonality (bio15), (c) average isothermality (bio3) (d) mean growing season temperature based on TREELIM (gst), (e) number of snow cover days (scd). The X axis in a–e shows the numeric sampling site, which are numbered from north (site 1) to south (site 20). Sites are also individually colored for direct comparison with plot (f) PCA biplot of all five climate conditions at individual tree coordinates, using the aforementioned shorthand codes to denote the 5 climate variables.
**Figure S2:**. Pearson correlations among geography, environment, and genetic structure for sampled trees. Geographic characteristics (latitude and longitude), 5 chosen environmental variables, and the first 3 genetic principal components of the SNP dataset (PC1‐3) as calculated with the vegan::rda() command. Graphs below the diagonal show scatter plots, the diagonal shows within‐parameter histograms, and values above the diagonal are correlation values.
**Figure S3:** Cumulative importance graph from Gradient Forest. U1‐U3 are population structure vectors from LFMM. Bio15 = prec.seasonality, bio3 = isothermality, scd = snow cover days, gst = growing season temperature, bio12 = annual precipitation.
**Figure S4:** Relationships between Gradient Forest offset values at sampling sites and site latitude across the three emission scenarios (rows) and two time frames (columns) by relative elevation class. Relative elevation class is indicated by point shape (■ = low, ◆ = middle, ▲ = high, ❋ = ungrouped) as well as color for pattern clarity (pink = high, blue = low, grey = middle and ungrouped). Solid regression lines indicate those with significant relationships (*p* ≤ 0.05), dashed indicate borderline significance (0.05 ≤ *p* ≤ 0.1), and missing lines indicate no meaningful relationship (*p* > 0.1). Line color matches elevation class (pink = high elevation; no low‐elevation correlations were significant so lines were omitted). Graphs with lines also show *R* ^2^ and *p*‐values of correlations.
**Figure S5:**. Relationships between LFMM offset values at sampling sites and site latitude across the three emission scenarios (rows) and two time frames (columns). Relative elevation class is indicated by point shape (■ = low, ◆ = middle, ▲ = high, ❋ = ungrouped) as well as color for pattern clarity (pink = high, blue = low, grey = middle and ungrouped). Solid regression lines indicate those with *p* ≤ 0.05, dashed with *p* ≤ 0.1, and missing line indicates no meaningful relationship (*p* > 0.1), with line color matching elevation class (i.e., pink = high elevation). Graphs with regression lines also show R2 and *p*‐values of correlations. No low‐elevation correlations had a significant relationship and lines were therefore omitted.
**Figure S6:** Relationships between Gradient Forest offset values at each sampling site and site latitude across the three emission scenarios (rows) and two time frames (columns) without relative elevation separation. Point shape indicates elevation class within a site pair (■ = low, ◆ = middle, ▲ = high) or ungrouped status (❋). Color indicates sampling area. Significant relationships (*p* ≤ 0.05) are indicated with a solid line, and marginally significant (*p* ≤ 0.1) with a dashed line, with R2 and *p*‐values for each relationship listed in the respective graphs.
**Figure S7:**. Relationships between Gradient Forest genomic offset values and latitude across *N. pumilio* range in Argentina, for all scenarios. Color indicates binned elevation. *R* ^2^ value and *p*‐values for each black linear regression line are shown at top right of each graph.
**Figure S8:**. Relationships between LFMM genomic offset values and latitude across *N. pumilio* range in Argentina, for all scenarios. Color indicates binned elevation. *R* ^2^ value and *p*‐values for each black linear regression line are shown at top right of each graph.
**Figure S9:**. Relationships between Gradient Forest genomic offset values, elevation, and latitude across *N. pumilio* range in Argentina, for all scenarios. Color indicates Gradient Forest Genomic Offset results, binned into 8 classes for readability and for comparison with true maps (e.g., Figure S12).
**Figure S10:**. Relationships between LFMM genomic offset values, elevation, and latitude across *N. pumilio* range in Argentina, for all scenarios. Color indicates LFMM Offset results, binned into 10 classes for readability and for comparison with true maps (e.g., Figure S11).
**Figure S12:**. Spatial projection map of Gradient Forest genomic offset values for the late‐century (2071–2100) and moderate‐emission scenario (SSP 3.70) for four representative areas of the *Nothofagus pumilio* range in Argentina. The shown areas each contain one sampling site pair, from north to south: (A) Cerro Otto (sites 4 & 5), (B) Lago Guacho (10–11), (C) El Calafate (17–18), and (D) Tierra del Fuego (19–20). Shapes indicate the elevation class of each site: high (▲) or low (⬛). Color of each 1‐km‐square pixel indicates the binned, projected Gradient Forest genomic offset value, from low (yellow/orange) to high (purple/black) (color scale same as Figure 3). Elevation isohypses indicate local topography, with thicker lines shown at 1000 and 2000 m a.s.l and thinner at every 200 m in between.
**Figure S13:**. Spatial projection map of LFMM genomic offset values for the late‐century (2071–2100) and moderate‐emission scenario (SSP 3.70) for four representative areas of the *Nothofagus pumilio* range in Argentina. The shown areas each contain one sampling site pair, from north to south: (A) Cerro Otto (sites 4 & 5), (B) Lago Guacho (10–11), (C) El Calafate (17–18), and (D) Tierra del Fuego (19–20). Shapes indicate the elevation class of each site: high (▲) or low (⬛). Color of each 1‐km‐square pixel indicates the binned, projected LFMM genomic offset value, from low (yellow/green) to high (blue/black) (color scale same as Figure S11). Elevation isohypses indicate local topography, with thicker lines shown at 1000 and 2000 m a.s.l and thinner at every 200 m in between.
**Table S1:** Locally common alleles and private alleles per the 11 sampling areas.
**Table S2:** Annotations for all genes containing SNPs that significantly associated (*q*‐value > 0.01) with multivariate climate space and were not previously identified by GEA methods.

## Data Availability

The SNP data used in this study have already been published by Sekely et al. (2024). Scripts used for genomic offset and graphing are available at: https://github.com/jtsekely/Nothofagus_genomic_offset.
