# Supplementary material for: A Mosaic of Future Maladaptation Predicted for the Widespread Tree Nothofagus pumilio
Source: Evol Appl. 2026 Mar 27;19(3):e70227. doi: 10.1111/eva.70227 (PMC13093351; doi:10.1111/eva.70227)
Supplement: Supplementary file 1 — Figure S1: Average annual climate conditions for each tree within each of the 20 sampling sites for Nothofagus pumilio based on CHELSA v2.1 for the reference period 1981–2010. Variables are (a) annual precipitation (shorthand: bio12), (b) precipitation seasonality (bio15), (c) average isothermality (bio3) (d) mean growing season temperature based on TREELIM (gst), (e) number of snow cover days (scd). The X axis in a–e shows the numeric sampling site, which are numbered from north (site 1) to south (site 20). Sites are also individually colored for direct comparison with plot (f) PCA biplot of all five climate conditions at individual tree coordinates, using the aforementioned shorthand codes to denote the 5 climate variables. Figure S2:. Pearson correlations among geography, environment, and genetic structure for sampled trees. Geographic characteristics (latitude and longitude), 5 chosen environmental variables, and the first 3 genetic principal components of the SNP dataset (PC1‐3) as calculated with the vegan::rda() command. Graphs below the diagonal show scatter plots, the diagonal shows within‐parameter histograms, and values above the diagonal are correlation values. Figure S3: Cumulative importance graph from Gradient Forest. U1‐U3 are population structure vectors from LFMM. Bio15 = prec.seasonality, bio3 = isothermality, scd = snow cover days, gst = growing season temperature, bio12 = annual precipitation. Figure S4: Relationships between Gradient Forest offset values at sampling sites and site latitude across the three emission scenarios (rows) and two time frames (columns) by relative elevation class. Relative elevation class is indicated by point shape (■ = low, ◆ = middle, ▲ = high, ❋ = ungrouped) as well as color for pattern clarity (pink = high, blue = low, grey = middle and ungrouped). Solid regression lines indicate those with significant relationships (p ≤ 0.05), dashed indicate borderline significance (0.05 ≤ p ≤ 0.1), and missing lines indicate no [file EVA-19-e70227-s001.zip › eva70227-sup-0001-FigureS1-S13@Sekely_ea_2025_GO_Supplemental_r1.docx]

Supplemental Materials for “A mosaic of maladaptation hotspots predicted for the widespread tree *Nothofagus pumilio”*

#
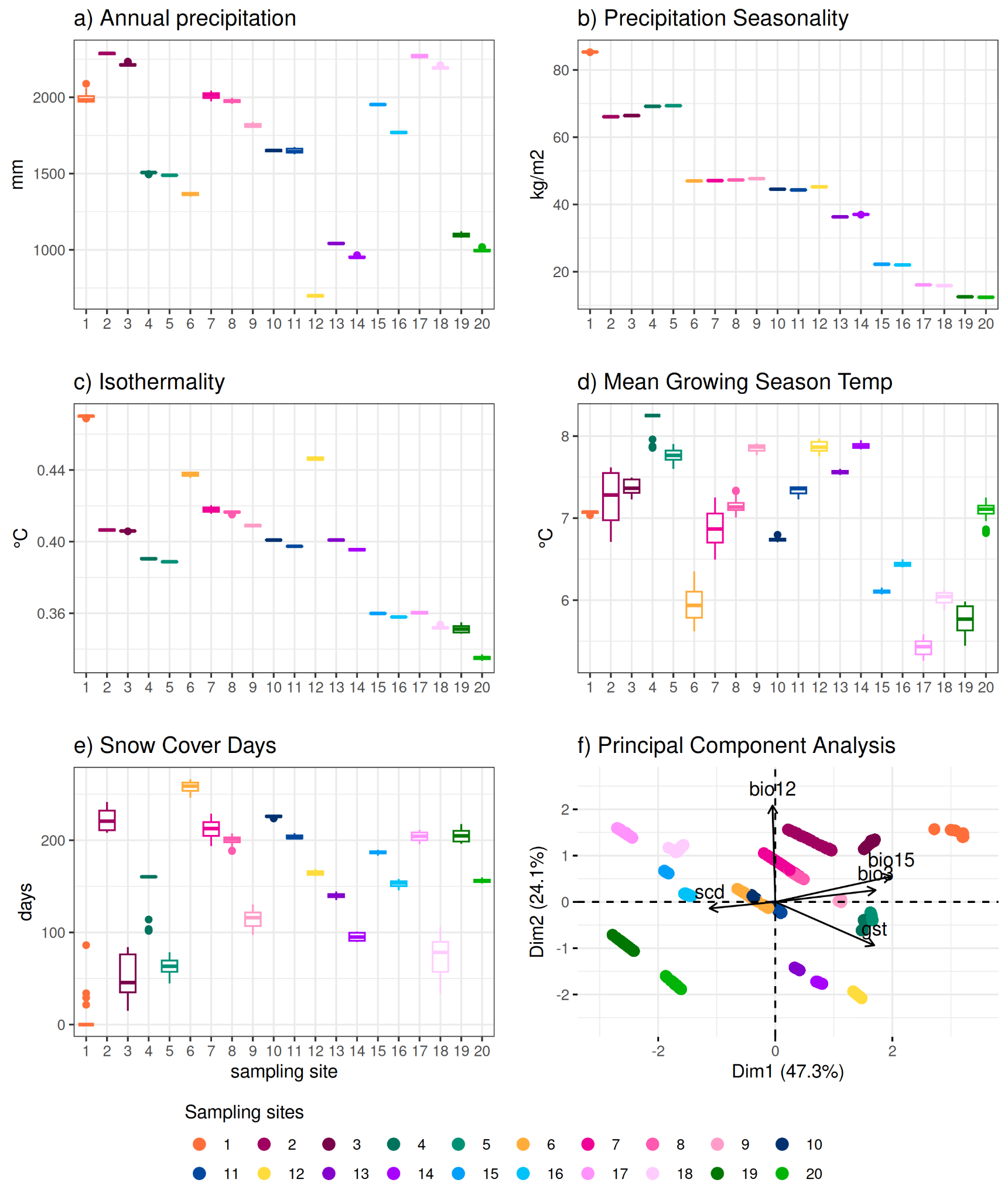


**Supplementary Figure 1.** Average annual climate conditions for each tree within each of the 20 sampling sites for *Nothofagus pumilio* based on CHELSA v2.1 for the reference period 1981-2010**.** Variables are a) annual precipitation (shorthand: bio12), b) precipitation seasonality (bio15), c) average isothermality (bio3) d) mean growing season temperature based on TREELIM (gst), e) number of snow cover days (scd). The X axis in a-e shows the numeric sampling site, which are numbered from north (site 1) to south (site 20). Sites are also individually colored for direct comparison with plot f) PCA biplot of all five climate conditions at individual tree coordinates, using the aforementioned shorthand codes to denote the 5 climate variables.


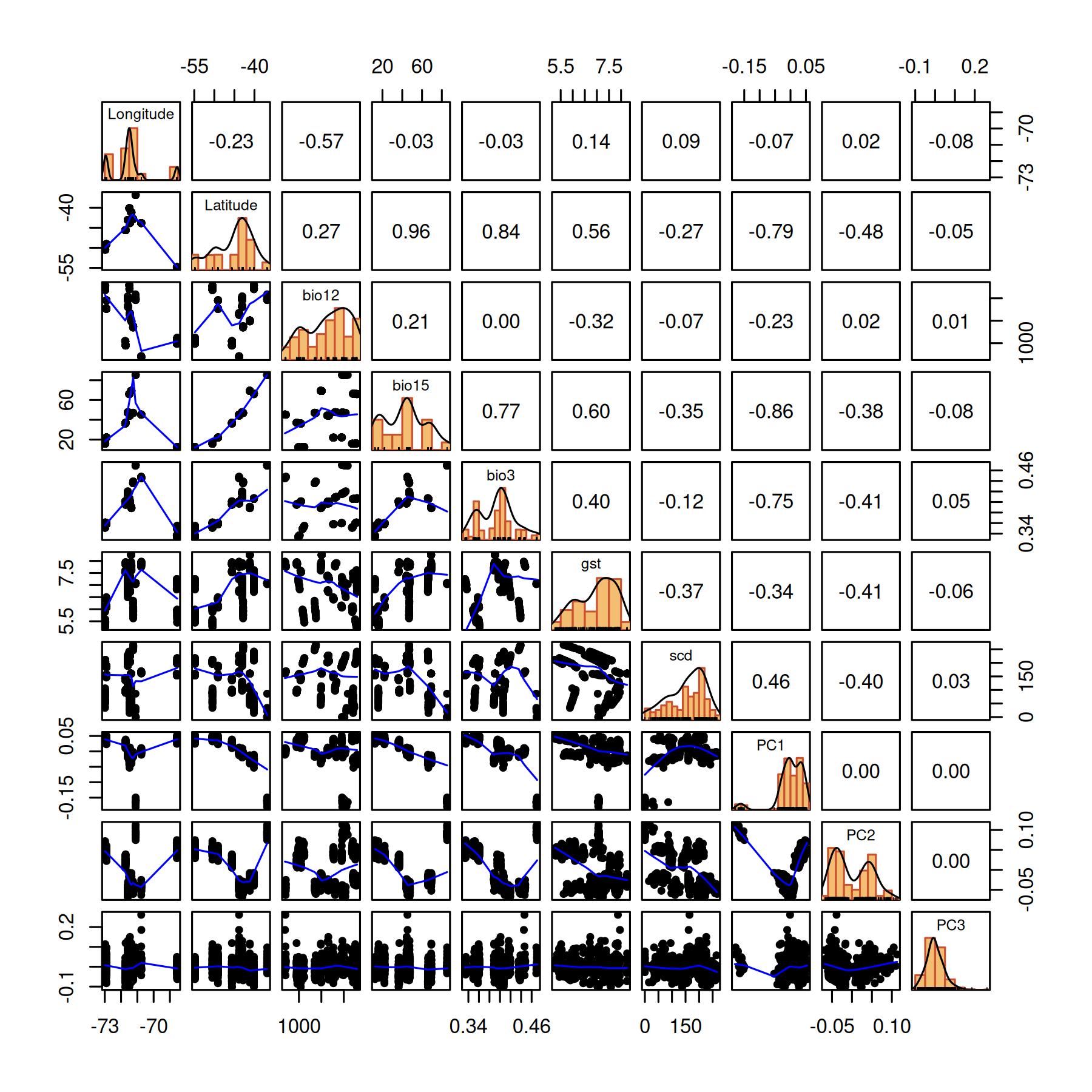


**Supplementary Figure 2.** Pearson correlations among geography, environment, and genetic structure for sampled trees. Geographic characteristics (latitude and longitude), 5 chosen environmental variables, and the first 3 genetic principal components of the SNP dataset (PC1-3) as calculated with the vegan::rda() command. Graphs below the diagonal show scatter plots, the diagonal shows within-parameter histograms, and values above the diagonal are correlation values.


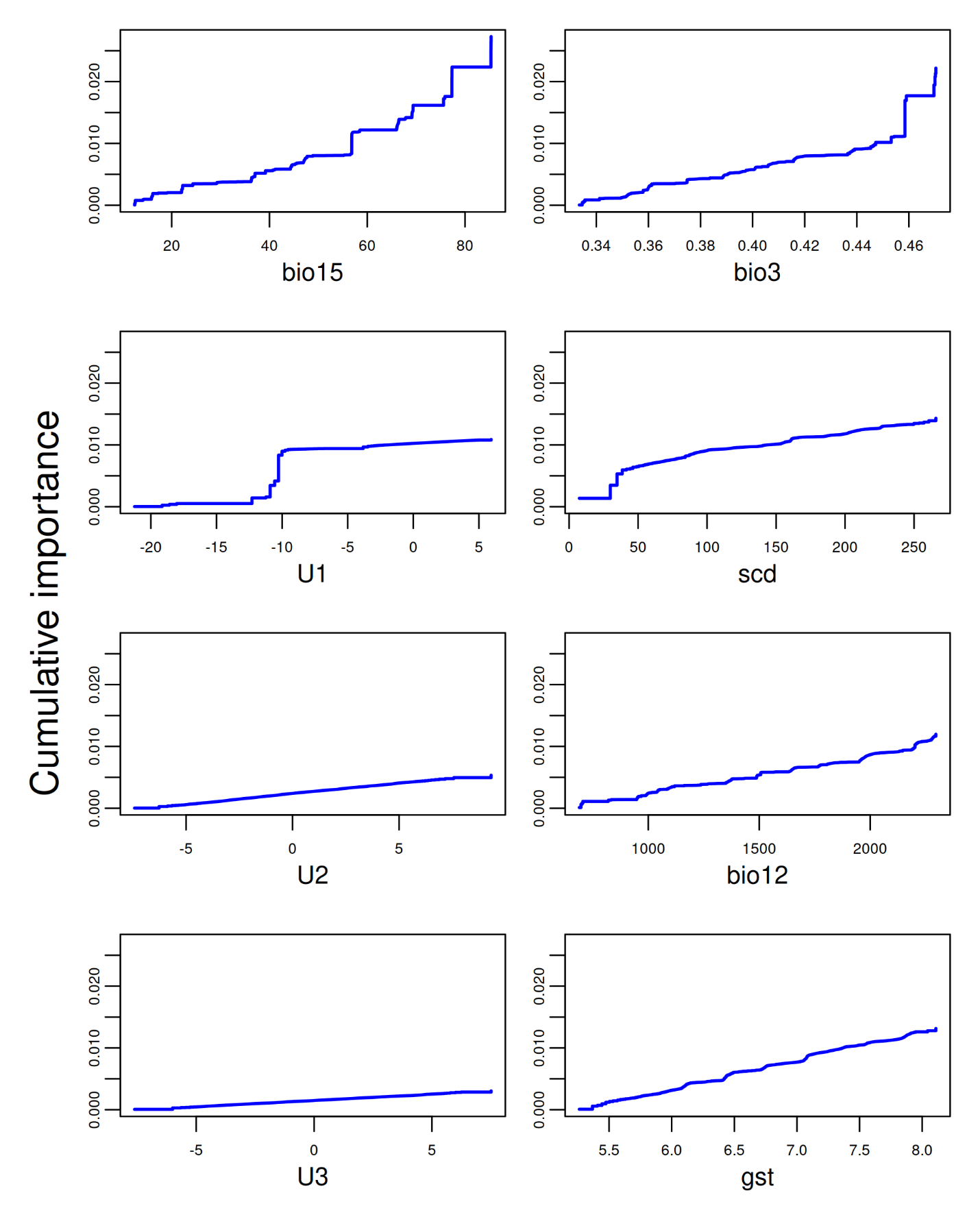


**Supplementary Figure 3.** Cumulative importance graph from Gradient Forest. U1-U3 are population structure vectors from LFMM. Bio15 = prec.seasonality, bio3 = isothermality, scd = snow cover days, gst = growing season temperature, bio12 = annual precipitation


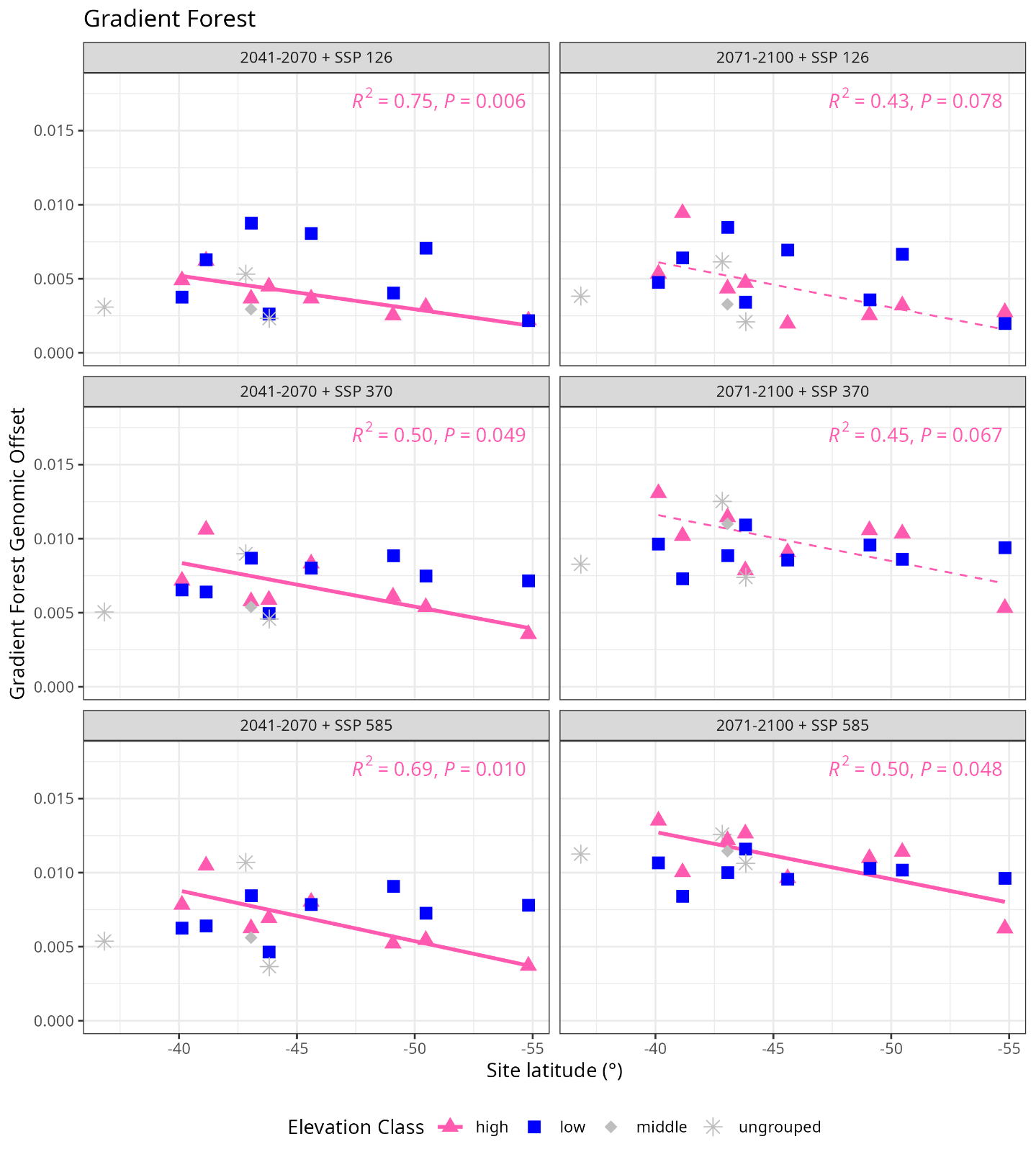


**Supplementary Figure 4.** Relationships between Gradient Forest offset values at sampling sites and site latitude across the three emission scenarios (rows) and two time frames (columns) by relative elevation class. Relative elevation class is indicated by point shape (■ = low, ◆ = middle, ▲ = high, ❋ = ungrouped) as well as color for pattern clarity (pink = high, blue = low, grey = middle and ungrouped). Solid regression lines indicate those with significant relationships (p ≤ 0.05), dashed indicate borderline significance (0.05 ≤ p ≤ 0.1), and missing lines indicate no meaningful relationship (p > 0.1). Line color matches elevation class (pink = high elevation; no low-elevation correlations were significant so lines were omitted). Graphs with lines also show R2 and p-values of correlations.


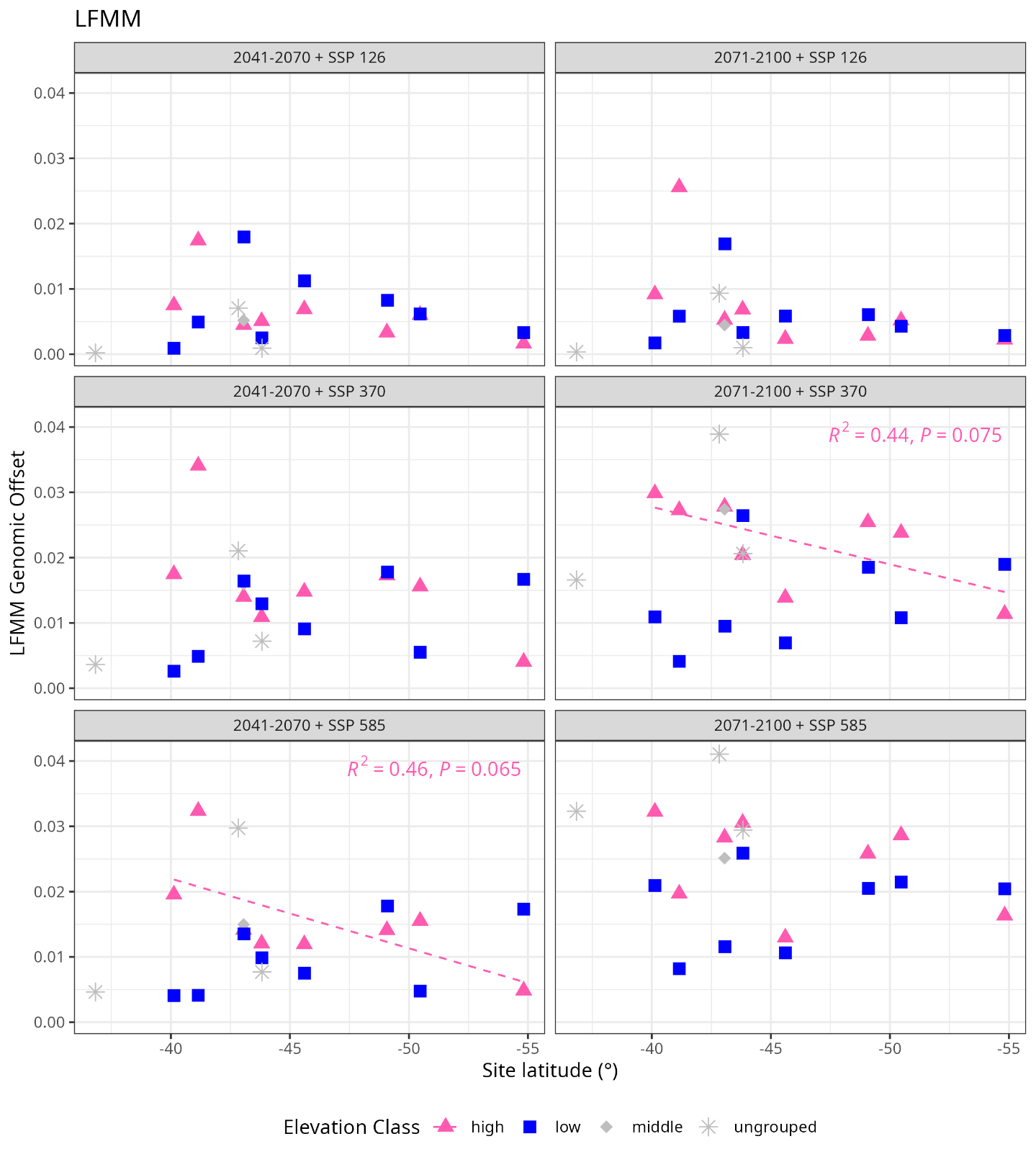


**Supplementary Figure 5**. Relationships between LFMM offset values at sampling sites and site latitude across the three emission scenarios (rows) and two time frames (columns). Relative elevation class is indicated by point shape (■ = low, ◆ = middle, ▲ = high, ❋ = ungrouped) as well as color for pattern clarity (pink = high, blue = low, grey = middle and ungrouped). Solid regression lines indicate those with p **≤** 0.05, dashed with p **≤** 0.1, and missing line indicates no meaningful relationship (p > 0.1), with line color matching elevation class (i.e. pink = high elevation). Graphs with regression lines also show R2 and p-values of correlations. No low-elevation correlations had a significant relationship and lines were therefore omitted.


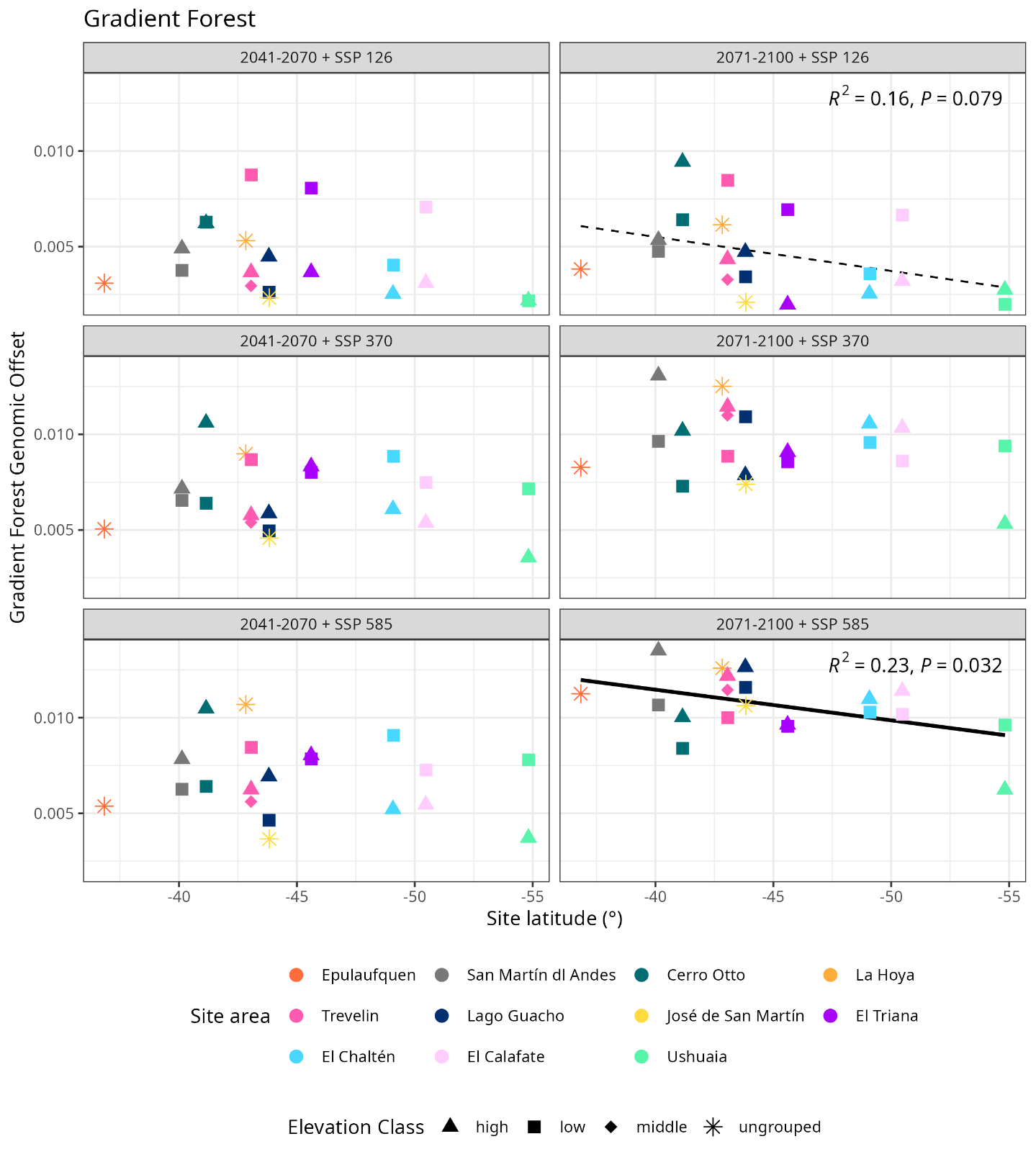


**Supplementary Figure 6. R**elationships between Gradient Forest offset values at each sampling site and site latitude across the three emission scenarios (rows) and two time frames (columns) without relative elevation separation. Point shape indicates elevation class within a site pair (■ = low, ◆ = middle, ▲ = high) or ungrouped status (❋). Color indicates sampling area. Significant relationships (p ≤ 0.05) are indicated with a solid line, and marginally significant (p ≤ 0.1) with a dashed line, with R2 and p-values for each relationship listed in the respective graphs.
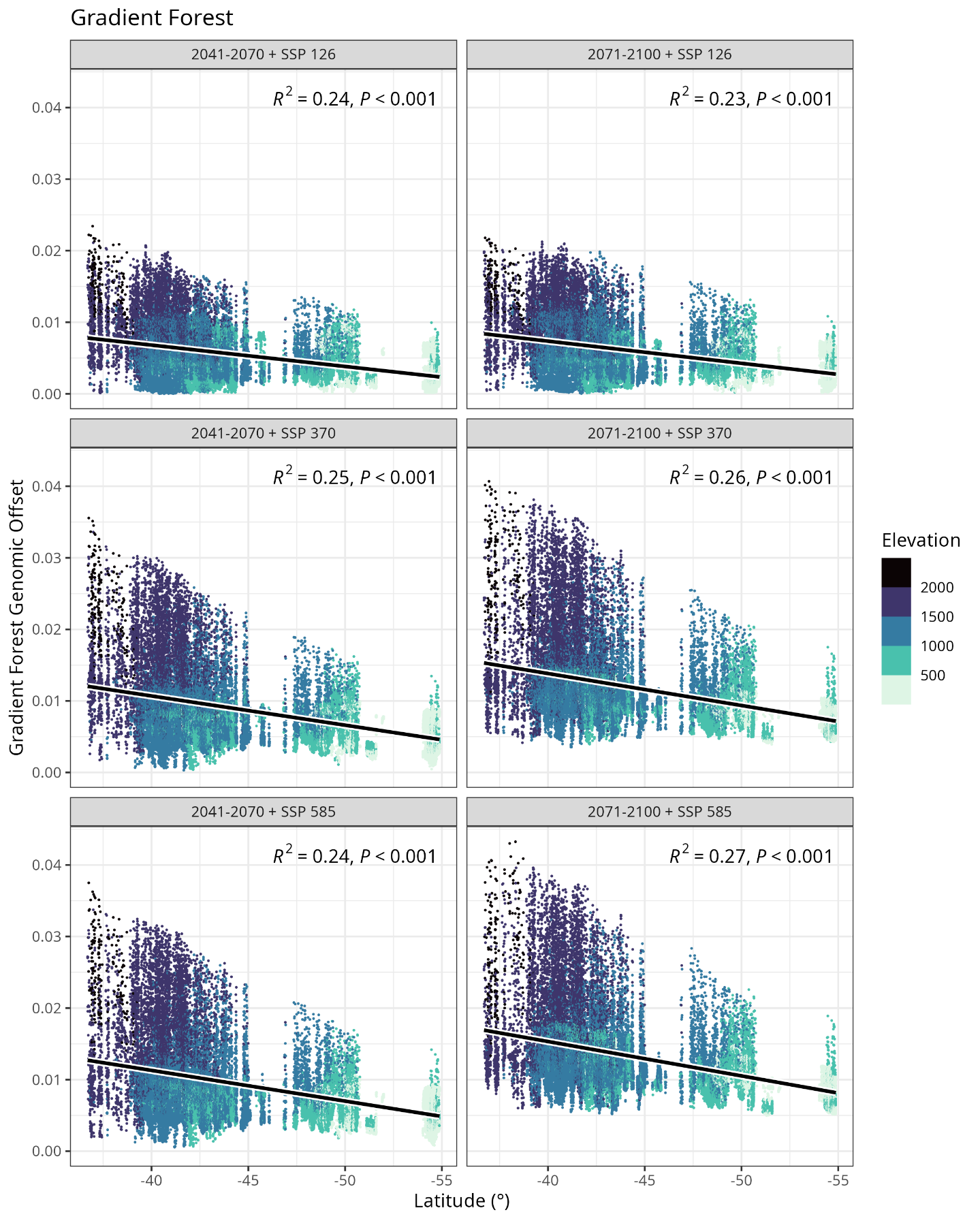


**Supplementary Figure 7.** Relationships between Gradient Forest genomic offset values and latitude across *N. pumilio* range in Argentina, for all scenarios. Color indicates binned elevation. R^2^ value and p-values for each black linear regression line are shown at top right of each graph.


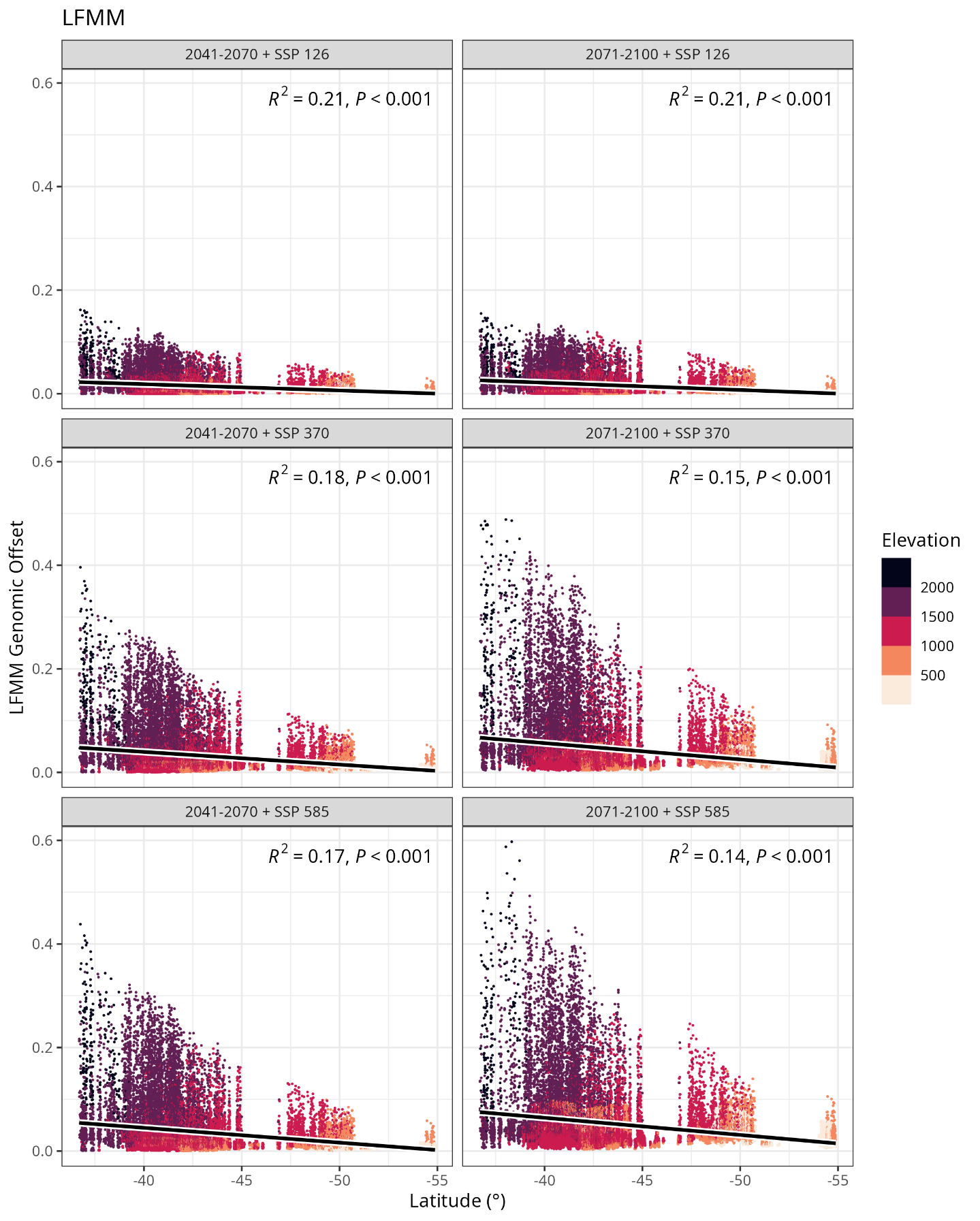


**Supplementary Figure 8.** Relationships between LFMM genomic offset values and latitude across *N. pumilio* range in Argentina, for all scenarios. Color indicates binned elevation. R^2^ value and p-values for each black linear regression line are shown at top right of each graph.


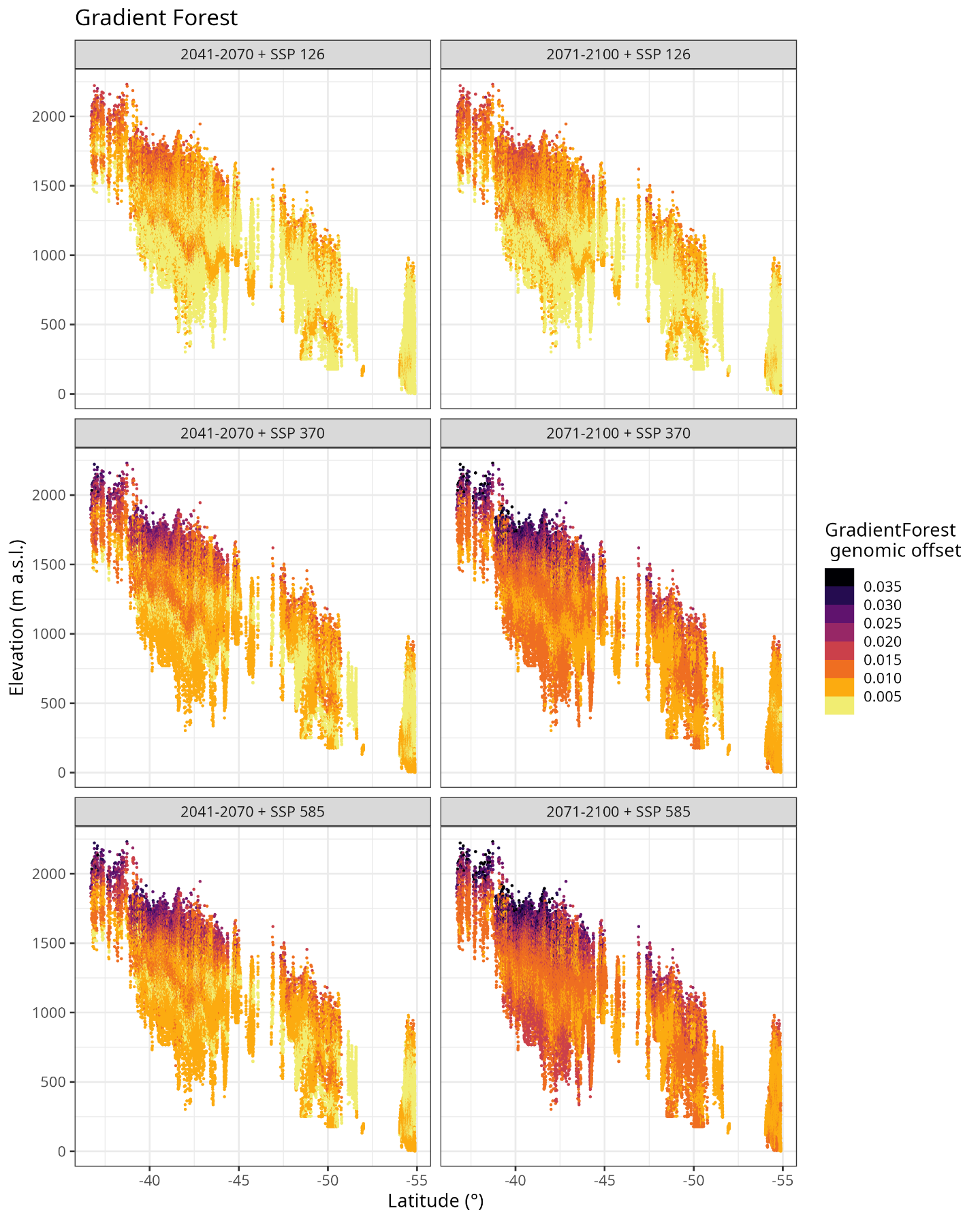


**Supplementary Figure 9.** Relationships between Gradient Forest genomic offset values, elevation, and latitude across *N. pumilio* range in Argentina, for all scenarios. Color indicates Gradient Forest Genomic Offset results, binned into 8 classes for readability and for comparison with true maps (e.g. Supplemental Figure 12).


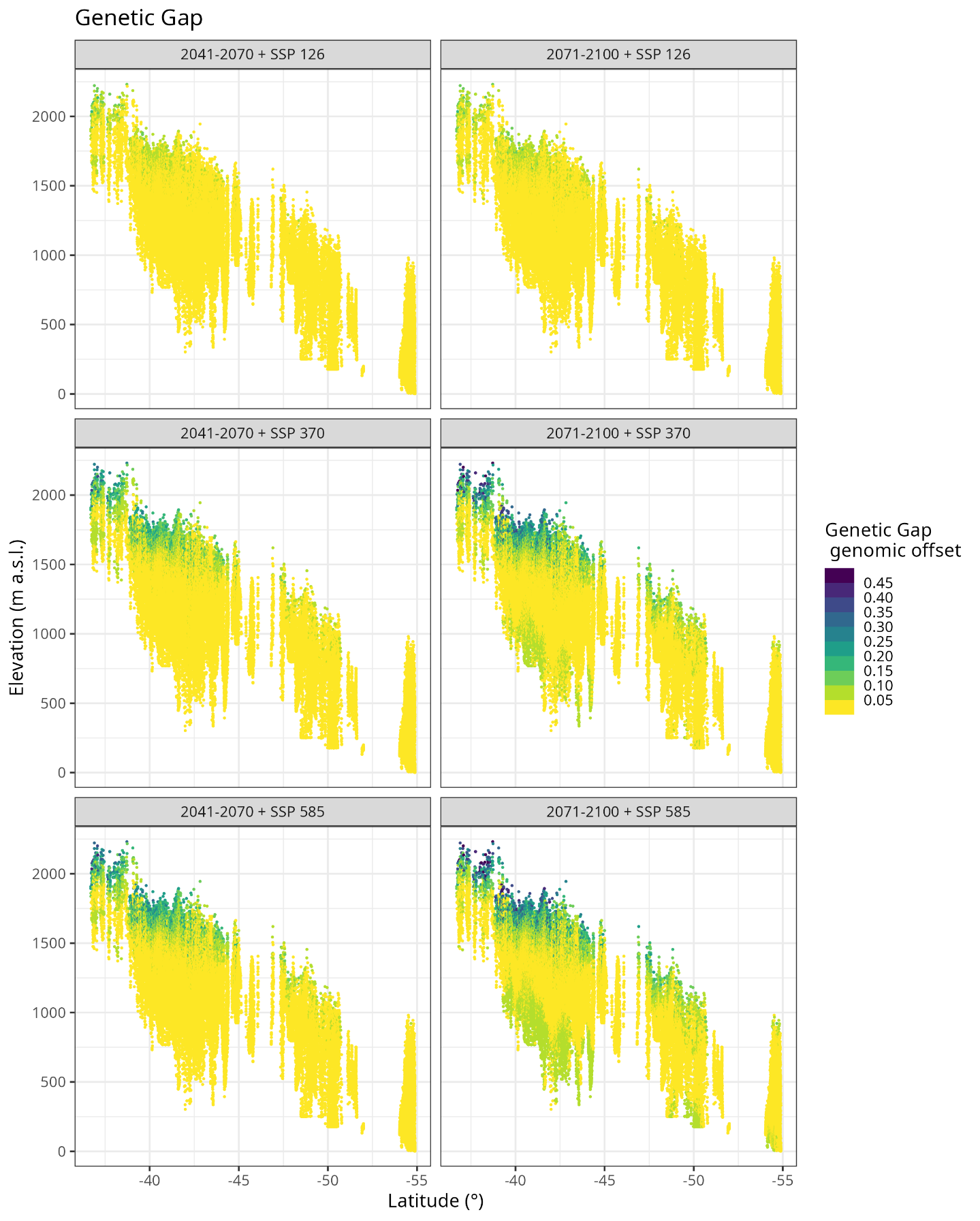


**Supplementary Figure 10.** Relationships between LFMM genomic offset values, elevation, and latitude across *N. pumilio* range in Argentina, for all scenarios. Color indicates LFMM Offset results, binned into 10 classes for readability and for comparison with true maps (e.g. Supplemental Figure 11)


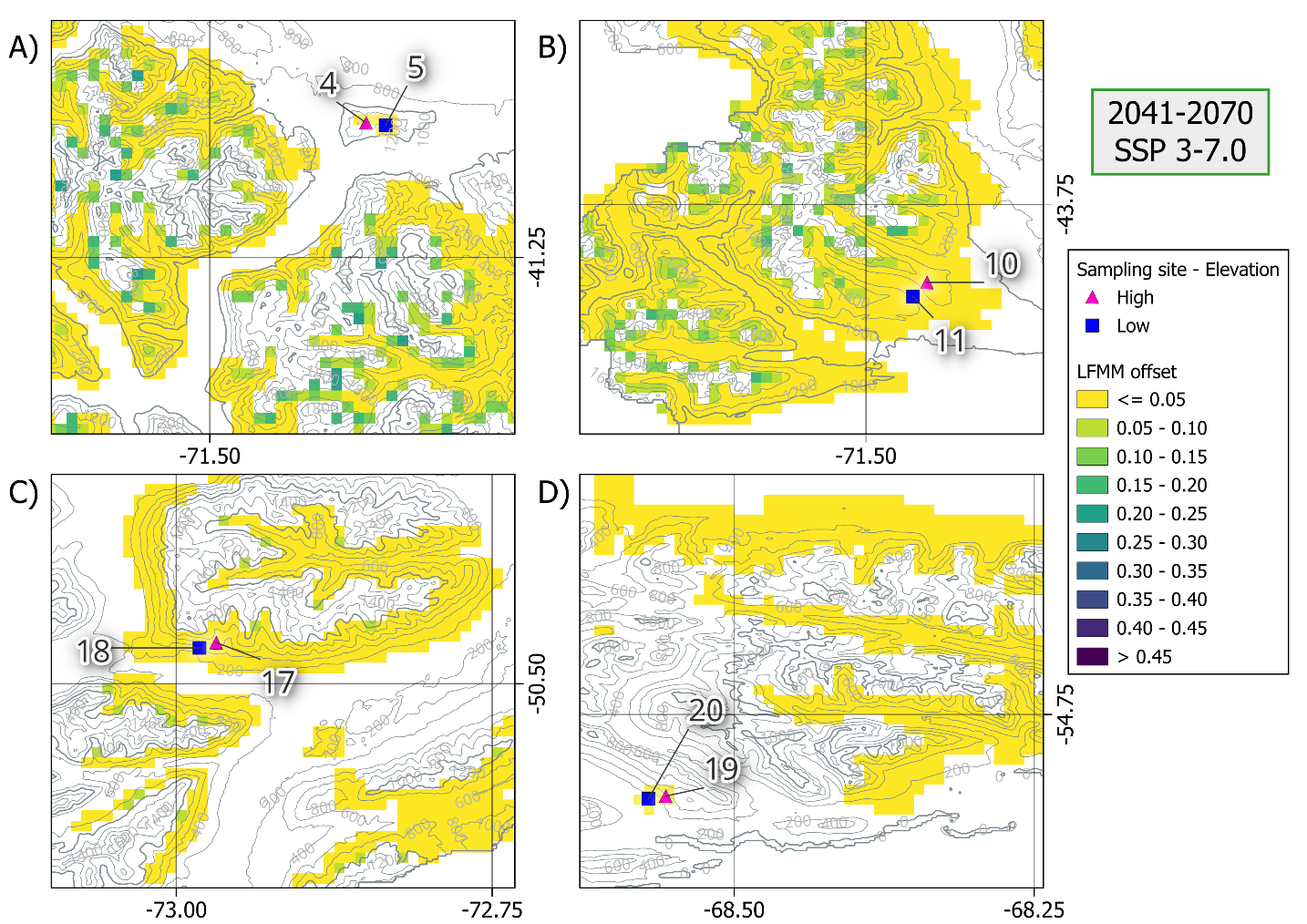
**Supplementary Figure 11.** Spatial projection map of LFMM genomic offset values for the mid-century (2041-2070) and moderate-emission scenario (SSP 3.70) for four representative areas of the *Nothofagus pumilio* range in Argentina. Complementary to Figure 3 in the manuscript. The shown areas each contain one sampling site pair, from north to south: A) Cerro Otto (sites 4 & 5), B) Lago Guacho (10-11), C) El Calafate (17-18), and D) Tierra del Fuego (19-20). Shapes indicate the elevation class of each site: high (▲) or low (⬛). Color of each 1-kilometer-square pixel indicates the binned, projected LFMM genomic offset value, from low (yellow/green) to high (blue/black) (colors intentionally differ from Figure 3 to reflect that values cannot be directly compared across methods). Elevation isohypses indicate local topography, with thicker lines shown at 1000 and 2000 meters a.s.l and thinner at every 200 m in between.


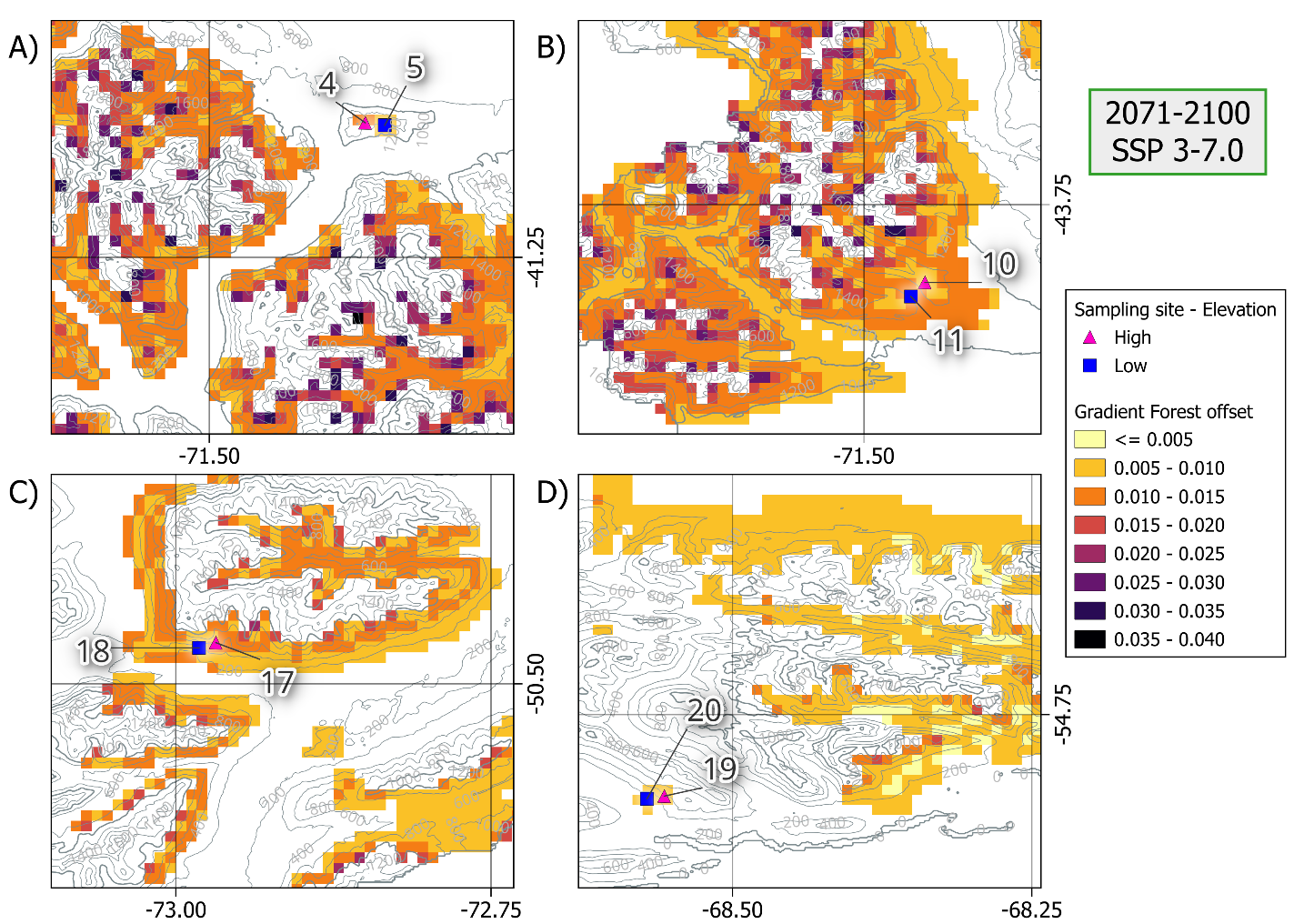


**Supplementary Figure 12.** Spatial projection map of Gradient Forest genomic offset values for the **late-century** (2071-2100) and moderate-emission scenario (SSP 3.70) for four representative areas of the *Nothofagus pumilio* range in Argentina. The shown areas each contain one sampling site pair, from north to south: A) Cerro Otto (sites 4 & 5), B) Lago Guacho (10-11), C) El Calafate (17-18), and D) Tierra del Fuego (19-20). Shapes indicate the elevation class of each site: high (▲) or low (⬛). Color of each 1-kilometer-square pixel indicates the binned, projected Gradient Forest genomic offset value, from low (yellow/orange) to high (purple/black) (color scale same as Figure 3). Elevation isohypses indicate local topography, with thicker lines shown at 1000 and 2000 meters a.s.l and thinner at every 200 m in between.


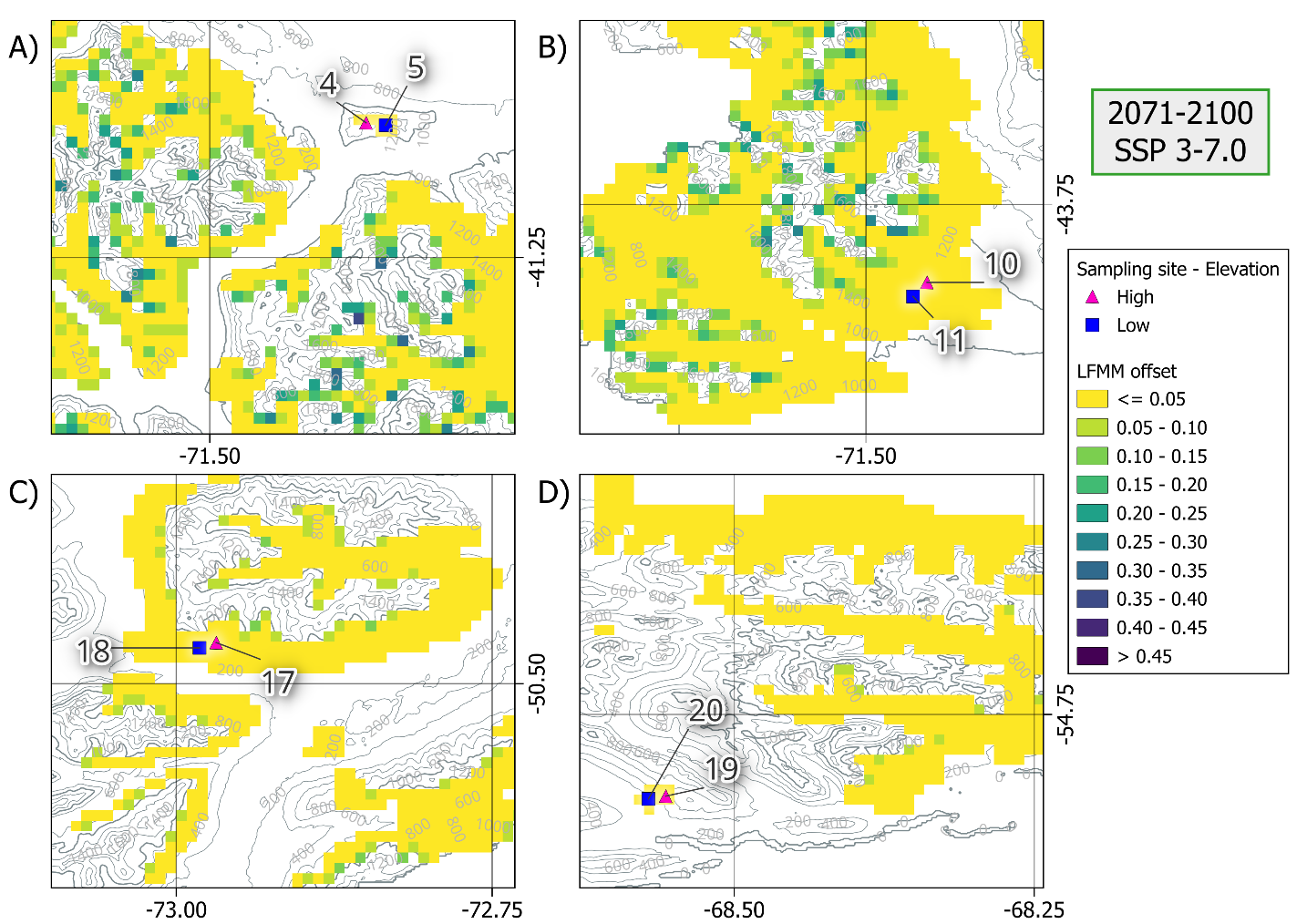


**Supplementary Figure 13**. Spatial projection map of LFMM genomic offset values for the **late-century** (2071-2100) and moderate-emission scenario (SSP 3.70) for four representative areas of the *Nothofagus pumilio* range in Argentina. The shown areas each contain one sampling site pair, from north to south: A) Cerro Otto (sites 4 & 5), B) Lago Guacho (10-11), C) El Calafate (17-18), and D) Tierra del Fuego (19-20). Shapes indicate the elevation class of each site: high (▲) or low (⬛). Color of each 1-kilometer-square pixel indicates the binned, projected LFMM genomic offset value, from low (yellow/green) to high (blue/black) (color scale same as Supplementary Figure 11). Elevation isohypses indicate local topography, with thicker lines shown at 1000 and 2000 meters a.s.l and thinner at every 200 m in between.

## Tables

**Supplemental Table 1. Locally common alleles and private alleles per the 11 sampling areas.** Sampling area names are accompanied by the site numbers in parentheses. Private alleles (PA) are the sum of all alleles unique to that sampling area, including very rare alleles (no MAF filter). A locally common allele (LCA) is one with a frequency greater than 5% in one area and a frequency less than 5% in all other areas.

| **Sampling area** | **PA** | **LCA** |
| --- | --- | --- |
| Epulaufquen (1) | 5392 | 1631 |
| San Martin dl Andes (2-3) | 4774 | 155 |
| Cerro Otto (4-5) | 3636 | 292 |
| La Hoya (6) | 1142 | 445 |
| Trevelin (7-9) | 3124 | 153 |
| Lago Guacho (10-11) | 1533 | 178 |
| Jose de San Martin (12) | 801 | 398 |
| El Triana (13-14) | 1195 | 208 |
| El Chalten (15-16) | 819 | 234 |
| El Calafate (17-18) | 672 | 364 |
| Tierra del Fuego (19-20) | 902 | 245 |
