# Supplementary material for: A Mosaic of Future Maladaptation Predicted for the Widespread Tree Nothofagus pumilio
Source: Evol Appl. 2026 Mar 27;19(3):e70227. doi: 10.1111/eva.70227 (PMC13093351; doi:10.1111/eva.70227)
Supplement: Supplementary file 1 — Figure S1: Average annual climate conditions for each tree within each of the 20 sampling sites for Nothofagus pumilio based on CHELSA v2.1 for the reference period 1981–2010. Variables are (a) annual precipitation (shorthand: bio12), (b) precipitation seasonality (bio15), (c) average isothermality (bio3) (d) mean growing season temperature based on TREELIM (gst), (e) number of snow cover days (scd). The X axis in a–e shows the numeric sampling site, which are numbered from north (site 1) to south (site 20). Sites are also individually colored for direct comparison with plot (f) PCA biplot of all five climate conditions at individual tree coordinates, using the aforementioned shorthand codes to denote the 5 climate variables. Figure S2:. Pearson correlations among geography, environment, and genetic structure for sampled trees. Geographic characteristics (latitude and longitude), 5 chosen environmental variables, and the first 3 genetic principal components of the SNP dataset (PC1‐3) as calculated with the vegan::rda() command. Graphs below the diagonal show scatter plots, the diagonal shows within‐parameter histograms, and values above the diagonal are correlation values. Figure S3: Cumulative importance graph from Gradient Forest. U1‐U3 are population structure vectors from LFMM. Bio15 = prec.seasonality, bio3 = isothermality, scd = snow cover days, gst = growing season temperature, bio12 = annual precipitation. Figure S4: Relationships between Gradient Forest offset values at sampling sites and site latitude across the three emission scenarios (rows) and two time frames (columns) by relative elevation class. Relative elevation class is indicated by point shape (■ = low, ◆ = middle, ▲ = high, ❋ = ungrouped) as well as color for pattern clarity (pink = high, blue = low, grey = middle and ungrouped). Solid regression lines indicate those with significant relationships (p ≤ 0.05), dashed indicate borderline significance (0.05 ≤ p ≤ 0.1), and missing lines indicate no [file EVA-19-e70227-s001.zip › eva70227-sup-0002-TableS2@Table_S2.pdf]

Table S2. Annotations for all genes containing SNPs that significantly associated (q-value > 0.01) with multivarite climate space and were not previously identified by GEA methods. *Nothofagus pumilio* locus is indicated by contig and pos[ition] and they are sorted alpha-numerically. P-values and q-values are shown for each. TAIR is the best-hit within *Arabidopsis thaliana*, and 'KEGG / GO' indicates the targeted ontology that was used to select this as a candidate gene.

Contig	Pos.	pvalue	qvalue	TAIR	Gene name	KEGG / GO
NODE_1320	1029	8.80E-05	0.004828	AT4G35985	Senescence/dehydration-associated protein-related	GO:0009409 (Response to cold)
NODE_1568	1880	0.000188	0.009161	NA	Unknown	NA
NODE_18812	580	2.39E-06	0.000283	At3g57450	Uncharacterized protein	NA
NODE_207	1496	0.000117	0.006215	AT5G07350	TUDOR-SN protein 1 (Tudor1, AtTudor1, TSN1)	GO:0034605 (cellular response to heat)
NODE_2265	295	1.99E-05	0.001578	AT2G26250	3-ketoacyl-CoA synthase 10 (FDH, KCS10)	Plant-pathogen interaction (KEGGmap04626) | GO:0009409 (Response to cold)
NODE_2515	1000	5.78E-05	0.003712	At2g20370	Xyloglucan galactosyltransferase MUR3 (EC 2.4.1.-) (Protein KATAMARI) (Protein MURUS 3) (AtMUR3) (Protein SHORT ROOT IN SALT MEDIUM 3)	endomembrane system organization [GO:0010256];fucose biosynthetic process [GO:0042353];protein glycosylation [GO:0006486];salicylic acid mediated signaling pathway [GO:0009863];unidimensional cell growth [GO:0009826];xyloglucan biosynthetic process [GO:0009969]
NODE_28521	315	0.000158	0.008004	NA	Unknown	NA
NODE_3377	162	2.49E-06	0.000289	AT5G08170	porphyromonas-type peptidyl-arginine deiminase family protein (EMB1873, ATAIH)	Arginine and proline metabolism (KEGGmap00330)
NODE_359	46	0.000167	0.008333	AT1G78230	Outer arm dynein light chain 1 protein	Yeaman (Consensus CG pine-spruce)
NODE_35935	163	0.000131	0.006847	NA	Unknown	NA
NODE_3632	150	0.000209	0.009907	AT5G05780	RP non-ATPase subunit 8A (RPN8A, AE3, ATHMOV34)	GO:0006955 (Immune response)
NODE_43113	126	6.69E-06	0.000662	AT1G50180	NB-ARC domain-containing disease resistance protein	GO:0006952 (Defense response)
NODE_45	1154	1.37E-05	0.001148	AT2G18790	phytochrome B (PHYB, HY3, OOP1)	Circadian rhythm (KEGGmap04712) | GO:0009409 (Response to cold)
NODE_503	2343	0.000126	0.006658	AT5G47910	respiratory burst oxidase homologue D (RBOHD, ATRBOHD)	MAPK signaling pathway (KEGGmap04016) | Plant-pathogen interaction (KEGGmap04626) | GO:0009408 (response to heat) | GO:0006952 (Defense response)
NODE_5405	923	0.000208	0.009873	AT1G16540	molybdenum cofactor sulfurase (LOS5) (ABA3) (SIR3, LOS5, ABA3, ATABA3, ACI2)	Stress ABA signalling pathway (PathwayStudio - TAIR) | GO:0009409 (Response to cold) | GO:0009408 (response to heat)
NODE_5764	71	1.32E-06	0.000172	AT2G30750	cytochrome P450, family 71, subfamily A, polypeptide 12 (CYP71A12)	Stilbenoid, diarylheptanoid and gingerol biosynthesis (KEGGmap00945) | GO:0006955 (Immune response)

NODE_5764	842	3.00E-05	0.002185	AT2G30750	cytochrome P450, family 71, subfamily A, polypeptide 12 (CYP71A12)	Stilbenoid, diarylheptanoid and gingerol biosynthesis (KEGGmap00945) | GO:0006955 (Immune response)
NODE_7325	908	6.10E-05	0.003881	AT3G28480	Oxoglutarate/iron-dependent oxygenase	Arginine and proline metabolism (KEGGmap00330)
NODE_7718	130	5.66E-05	0.003673	AT4G11150	vacuolar ATP synthase subunit E1 (TUF, emb2448, TUFF, VHA-E1)	GO:0009409 (Response to cold)
NODE_8149	611	6.22E-05	0.003929	AT2G23620	methyl esterase 1 (ATMES1, MES1)	GO:0006955 (Immune response)
NODE_8149	963	3.05E-05	0.002203	AT2G23620	methyl esterase 1 (ATMES1, MES1)	GO:0006955 (Immune response)
TOC_TOC	1828	5.80E-05	0.003712	AT5G61380	CCT motif -containing response regulator protein (TOC1, APRR1, PRR1, AtTOC1)	Circadian rhythm (KEGGmap04712)
chain_11196	826	7.93E-05	0.004507	AT1G19100	Histidine kinase-, DNA gyrase B-, and HSP90-like ATPase family protein	RNA-directed DNA methylation | GO:0006952 (Defense response)
chain_11196	2170	6.28E-05	0.00394	AT1G19100	Histidine kinase-, DNA gyrase B-, and HSP90-like ATPase family protein	RNA-directed DNA methylation | GO:0006952 (Defense response)
chain_11318	1743	3.94E-05	0.002701	AT5G18650	CHY-type/CTCHY-type/RING-type Zinc finger protein	GO:0006952 (Defense response)
chain_11318	3427	0.000135	0.006956	AT5G18650	CHY-type/CTCHY-type/RING-type Zinc finger protein	GO:0006952 (Defense response)
chain_1133	990	0.000161	0.008111	AT5G05340	Peroxidase superfamily protein	Phenylpropanoid biosynthesis (KEGGmap00940)
chain_11645	1870	3.36E-05	0.002371	AT5G11110	sucrose phosphate synthase 2F (SPS1, ATSPS2F, KNS2, SPS2F)	Starch and sucrose metabolism (KEGGmap00500)
chain_12019	3525	9.60E-06	0.000887	AT1G47840	hexokinase 3 (HXK3)	Starch and sucrose metabolism (KEGGmap00500) | GO:0009409 (Response to cold)
chain_12401	1157	4.78E-05	0.00321	AT5G61040	Uncharacterized protein	NA
chain_12836	273	0.000198	0.009534	AT5G58090	O-Glycosyl hydrolases family 17 protein	Starch and sucrose metabolism (KEGGmap00500) | GO:0006952 (Defense response)
chain_12853	493	5.14E-05	0.003403	AT5G04660	cytochrome P450, family 77, subfamily A, polypeptide 4 (CYP77A4)	Stilbenoid, diarylheptanoid and gingerol biosynthesis (KEGGmap00945)
chain_1800	187	8.77E-05	0.004828	AT1G32900	UDP-Glycosyltransferase superfamily protein	Starch and sucrose metabolism (KEGGmap00500)
chain_1800	214	6.54E-05	0.003999	AT1G32900	UDP-Glycosyltransferase superfamily protein	Starch and sucrose metabolism (KEGGmap00500)
chain_18733	712	7.78E-06	0.000754	AT4G31140	O-Glycosyl hydrolases family 17 protein	Starch and sucrose metabolism (KEGGmap00500) | GO:0006952 (Defense response)
chain_3182	499	0.000177	0.008762	AT3G49680	branched-chain aminotransferase 3 (ATBCAT-3, BCAT3)	Glucosinolate biosynthesis (KEGGmap00966)
chain_3420	2219	0.000133	0.006912	AT3G12250	TGACG motif-binding factor 6 (TGA6, BZIP45)	Plant hormone signal transduction (KEGGmap04075) | GO:0006955 (Immune response)
chain_54947	2179	1.07E-05	0.000954	NA	Unknown	NA

chain_5907	4689	8.42E-05	0.004701	At4g23400	Probable aquaporin PIP1-5 (AtPIP1;5) (Plasma membrane intrinsic protein 1d) (PIP1d)	response to water deprivation [GO:0009414]
chain_5996	1097	6.44E-06	0.000658	At3g17650	Probable metal-nicotianamine transporter YSL5 (Protein YELLOW STRIPE LIKE 5) (AtYSL5)	NA
chain_6678	1037	7.76E-05	0.004462	AT4G17750	heat shock factor 1 (HSF1, HSFA1A, ATHSF1, ATHSFA1A)	GO:0009408 (response to heat)
chain_7641	2137	6.26E-06	0.000653	AT2G45660	AGAMOUS-like 20 (AGL20, SOC1, ATSOC1)	Flowering genes not included in KEGGmap04712 | GO:0009409 (Response to cold)
chain_7839	213	0.000199	0.009534	AT1G19840	SAUR-like auxin-responsive protein family	Plant hormone signal transduction (KEGGmap04075)
chain_8002	2754	6.73E-05	0.004022	AT3G18165	modifier of snc1,4 (MOS4)	GO:0006955 (Immune response)
chain_914	691	6.63E-05	0.004004	At3g62290	ADP-ribosylation factor	intracellular protein transport [GO:0006886];vesicle-mediated transport [GO:0016192]
